# Simulation-guided biomimetic sharp-edged ultrasonic microreactor enables morphology-uniform and performance-tunable halide perovskite quantum dots

**DOI:** 10.1016/j.ultsonch.2026.107948

**Published:** 2026-07-03

**Authors:** Shengxin Zhu, Jianwei Liao, Longshi Rao, Yuying Wang, Xiang Huo, Qinghao Zhong, Haoyu Chen, Guisheng Zhong, Xiaodong Niu

**Affiliations:** a Department of Mechanical Engineering, College of Engineering, Shantou University, Shantou 515063, China; b Intelligent Manufacturing Key Laboratory of Ministry of Education, Shantou University, Shantou 515063, China; c Guangdong Provincial Key Laboratory of Automotive Display and Touch Technologies, Shantou Goworld Display Technology Co., Ltd., Shantou 515041, China; d Shantou Key Laboratory for Intelligent Equipment and Technology, Shantou University, Shantou 515063, China

**Keywords:** Halide perovskite quantum dots, Sharp-edged microstructures, Ultrasonic microreactor, Ultrasonic cavitation effect

## Abstract

Halide perovskite quantum dots (HPQDs) are transformative candidates for next-generation optoelectronic devices, owing to their exceptional optoelectronic properties including widely tunable bandgaps, ultrahigh color purity, and solution processability. However, scalable, deterministic synthesis of high-quality HPQDs with simultaneous ultra-narrow emission linewidth and high photoluminescence quantum yield (PLQY) remains a longstanding challenge, fundamentally limited by the mass transfer bottleneck and poor mixing efficiency of conventional laminar microreactors. Here, we report a biomimetic vein-inspired ultrasonic microreactor integrated with sharp-edged microstructure arrays to address this core challenge. Through systematic multiphysics simulations, we quantitatively decode the acoustic-hydrodynamic coupling mechanism in the microreactor, and establish a quantitative structure-performance relationship between microstructure geometry and sonochemical reaction performance. We identify an optimized cylindrical microstructure configuration that synergistically amplifies acoustic streaming and cavitation yield to break laminar boundary layer confinement. Experimental validation confirms the optimized microreactor enables continuous synthesis of high-quality HPQDs with an ultra-narrow full width at half maximum of 23.28 nm and PLQY up to 78.6%, markedly outperforming conventional microfluidic methods. We further elucidate that cavitation-enhanced micromixing enables dynamic supersaturation tuning, driving LaMer-type size-focusing and homogeneous nucleation for exceptional HPQDs monodispersity. This work provides a generalizable, scalable microfluidic strategy for precision synthesis of high-performance optoelectronic nanomaterials, bridging the critical gap between lab-scale research and industrial translation.

## 1 Introduction

Halide perovskite quantum dots (HPQDs) are emerging as transformative candidates for next-generation optoelectronic devices — including ITU-R Rec. BT.2020-compliant display [1], [2], [3], high-efficiency photodetectors, and high-resolution biomedical imaging — owing to their unique combination of high carrier mobility, tunable bandgap, and exceptional photoluminescence quantum yield (PLQY) [4], [5], [6], [7], [8]. Critically, the potential of these HPQDs-enabled applications is fundamentally contingent on material optical quality, with spectral purity (defined by ultra-narrow emission linewidths) and high PLQY serving as paramount determinants [9], [10], [11]. Consequently, the synthesis of high-quality HPQDs characterized by ultra-narrow full width at half maximum (FWHM, typically < 30 nm) and high PLQY is not merely fundamental scientific pursuit but also a critical industrial bottleneck that must be overcome to enable successful commercialization [12], [13], [14], [15]. Achieving these stringent performance metrics directly dictates the operational efficacy of HPQDs-based devices, particularly in terms of color fidelity for display technologies [16], [17]. Accordingly, developing a robust, scalable methodology capable of transcending the inherent limitations of current solution-based or vapor-phase synthesis approaches — such as batch-to-batch inconsistency and poor morphological uniformity — to stably produce HPQDs with superior optical signatures has become a defining focus in advanced chemical engineering [18].

Despite their tremendous potential, the ionic crystal lattice inherent to HPQDs drives extremely fast nucleation and growth kinetics, posing significant challenges for improving morphological uniformity and achieving performance controllability during the synthesis process [19]. Conventional macroscale batch reactors, which rely on methods such as hot-injection or ligand-assisted re-precipitation, remain the dominant synthesis routes for HPQDs [20], [21], [22], [23], [24]. Critically, the inherent inefficiencies in mass and heat transfer within macrosystems — exacerbated by the poor mixing efficiency inherent to their structural design — frequently generate pronounced local supersaturation gradients [25], [26]. This inhomogeneous reactive microenvironment disrupts the delicate balance of crystal nucleation and growth kinetics, ultimately resulting in size polydispersity (> 30% in typical cases), broadened FWHM (> 50 nm), and significantly compromised optical performance, including reduced PLQY [27], [28]. Furthermore, the lack of batch-to-batch reproducibility and inherent difficulties in continuous scale-up further impede the translation of high-performance HPQDs from lab-scale synthesis to industrial-scale production and practical deployment [29], [30], [31].

Microfluidics has emerged as a transformative pathway for the continuous synthesis of nanomaterials, leveraging high surface-to-volume ratios (typically 10^3^–10^4^ m^−1^) and precise manipulation of reaction parameters [32], [33]. Nevertheless, traditional smooth-walled microchannels are restricted by a physical bottleneck: the dominance of laminar flow (Reynolds number *R*_*e*_ < 2300 in typical microfluidic systems) . In such fluidic environments, mixing relies predominantly on slow molecular diffusion (diffusion coefficients ∼ 10^−9^ m^2^/s for organic-inorganic precursors), which is kinetically mismatched with the ultra-rapid nucleation kinetics of HPQDs [34], [35]. This “passive mixing” mode fails to generate sufficient shear forces for rapid precursor dispersion, resulting in pronounced concentration gradients and inconsistent nucleation rates — key drivers of HPQDs polydispersity and degraded optical performance. Thus, synthesizing ideal HPQDs demands breaking laminar flow constraints and engineering controllable intense turbulence and localized high-energy cavitation zones at the microscale [31].

To address the limitations of diffusion-limited laminar flow in microfluidics, integration of external fields — particularly ultrasound — has been established as a powerful strategy for micromixing intensification [19], [36]. The core mechanism of this process intensification is acoustic cavitation induced by ultrasound in the fluid medium: when the negative pressure generated by ultrasonic waves exceeds the liquid’s tensile strength, microscopic cavitation bubbles rapidly nucleate, grow, and violently implode within microseconds. This transient phenomenon induces extreme local physical effects, including so-called “hot spots” (temperatures up to 5000 K, pressures exceeding 10 MPa), intense shock waves, and high-speed microjets. These high-energy effects generate immense shear forces that disrupt the laminar boundary layer to drastically enhance mass transfer, deagglomerate HPQDs clusters, and homogenize the reactive microenvironment. While integrating an external ultrasonic field can intensify mixing via acoustic cavitation, cavitation bubbles in conventional straight microchannels exhibit stochastic generation and uneven spatial intensity distribution (variations > 40% across the channel cross-section), hampering nucleation and growth over HPQDs crystal growth [37].

To address these limitations, we develop an ultrasonic microreactor integrated with sharp-edged microstructure arrays. Bioinspired by the efficient mass and energy transport mechanisms of fractal leaf veins — where hierarchical branching enables uniform fluid distribution and rapid mass transfer — these sharp-edged structures are rationally designed as dual-functional hydrodynamic modulators. Beyond disrupting the laminar boundary layer to induce intense local turbulence, they more critically function as preferential nucleation sites for acoustic cavitation, significantly lowering the cavitation threshold and directionally amplifying the release of ultrasonic energy. This innovative design enables a spatially uniform, high-energy reactive environment, ensuring synchronized nucleation and homogeneous growth of HPQDs, overcoming longstanding challenges in size polydispersity and optical quality.

Specifically, we performed numerical simulations using COMSOL Multiphysics to investigate the effects of sharp-edged structural parameters — including morphological features (tip angle, edge curvature, height, and inter-structure spacing) — on flow field characteristics and the spatiotemporal distribution of cavitation, thereby identifying the optimal microstructural configuration. Guided by these simulation results, we established an efficient, controllable experimental system that successfully yielded high-quality HPQDs with an narrow FWHM of 23.28 nm and a PLQY of 78.6% — performance metrics that are comparable to those of mainstream HPQDs synthesis strategies while offering markedly superior batch-to-batch reproducibility. Furthermore, we investigated the coupling effects of key process parameters (flow rate, precursor concentration, ultrasonic frequency) on the resulting HPQDs morphology and optical performance. This work elucidates the mechanisms of microstructure-induced acoustic cavitation enhancement, and provides a scalable strategy for high-performance perovskite nanomaterial synthesis, with broad implications for optoelectronic, photovoltaic, and biomedical applications.

## 2 Experimental section

### 2.1 Chemicals and materials

All chemical reagents used for HPQDs synthesis were of analytical grade or higher to ensure experimental reproducibility and consistency. Cesium bromide (CsBr, AR, ≥ 99.9%), lead bromide (PbBr_2_, AR, ≥ 99.9%), oleic acid (OA, AR), oleylamine (OAm, 80%–90%), and N, N-dimethylformamide (DMF, AR, ≥ 99.5%) were purchased from Shanghai Aladdin Biochemical Technology Co., Ltd. Chlorobenzene (ACS grade) and castor oil (USP grade) were sourced from Shanghai Macklin Biochemical Technology Co., Ltd. All reagents were used as received without further purification.

### 2.2 Structural design and optimization of sharp-edged vein microchannels

**Fig. 1:**
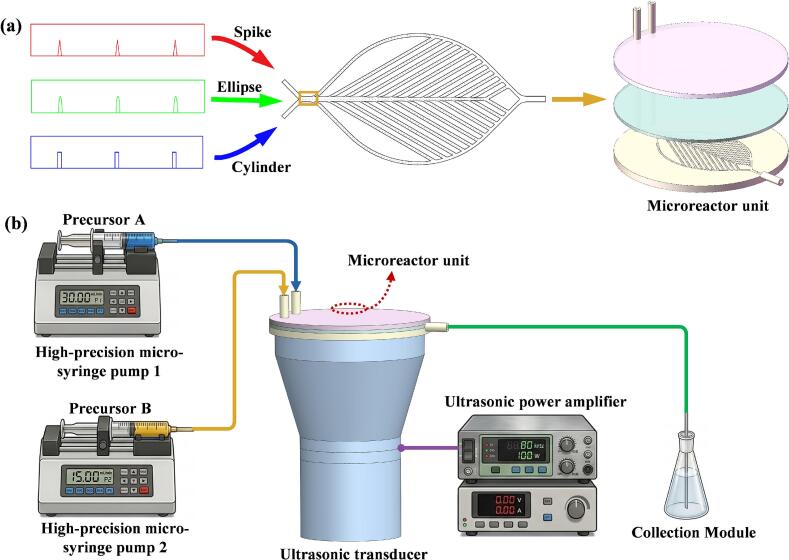
(a) Front view and exploded view of the vein-inspired microchannel integrated with sharp-edged microstructures, alongside schematics illustrating the morphological designs of three distinct microstructures: spiked, elliptical, and cylindrical configurations. (b) Schematic illustration of the component layout of the sharp-edged ultrasonic microreactor system, depicting the key functional modules of the integrated setup.

Inspired by the efficient transport mechanisms of water in natural leaf vein networks [18] — where hierarchical fractal branching and micro-nano textured surfaces enable uniform fluid distribution and rapid mass/energy transfer — we propose a bioinspired vein microchannel design integrated with periodic sharp-edged microstructures (Fig. S1). Natural leaf veins serve as an ideal biological template for microfluidic channel topological optimization, as their intrinsic architecture inherently addresses the limitations of conventional smooth-walled microchannels (e.g., poor mixing efficiency, uneven energy distribution). To replicate and enhance this natural high-efficiency transport performance, sharp-edged microstructures were strategically incorporated into the channel interior (Fig. 1a), arranged in a periodic linear array along the fluid flow direction to maximize structure-fluid-acoustic field interactions.

The core innovation of this design lies in the construction of a strong “structure-acoustic field” coupling effect. Under excitation by an external ultrasonic field, the geometrically regular sharp-edged structures act as “geometric singularities”, significantly amplifying the local acoustic pressure gradient and trigger the rapid nucleation, growth, and violent collapse of cavitation bubbles. This enhanced cavitation dynamics systematically perturbs the stagnant laminar boundary layer within the microchannel, substantially intensifying chaotic mixing, interfacial heat transfer, and particle manipulation capabilities. Through the precise parametric design of the geometric morphology, height (*H*), and spacing (*d*) of the sharp-edged microstructures, we achieve active and synergistic regulation of the hydrodynamic field, acoustic field, and chemical reaction concentration field within the microchannel, subsequently optimizing multiphase mass transfer efficiency and reaction kinetics.

### 2.3 Construction of the ultrasonic microreactor system

Fig. 1b illustrates the overall configuration of the sharp-edged ultrasonic microreactor system, which comprises four distinct functional modules: (1) Feed Module: Two high-precision micro-syringe pumps were used to ensure stable, controllable transport of reactant solutions into the microreactor, minimizing flow rate fluctuations that could perturb reaction kinetics. (2) Microreactor Module: This module featured a double-layer architecture, with a bottom layer embedded with the vein-inspired sharp-edged microchannels to guide uniform, high-efficiency fluid flow, and a top layer consisting of a smooth glass cover slip with two inlets and one outlet to form a closed fluidic pathway. Hermetic sealing between the two layers was achieved using a fluorinated ethylene propylene gasket to prevent reagent leakage and ensure reaction reproducibility. (3) Ultrasonic Control Module: Comprising an ultrasonic transducer, an ultrasonic amplifier, and the microreactor unit, this module generated and precisely regulated the ultrasonic field applied to the reaction medium, with real-time monitoring of acoustic power output. (4) Product Collection Module: Synthesized HPQDs samples were continuously collected in a pre-cleaned glass flask at the microreactor outlet, maintained at room temperature to avoid post-synthesis aggregation.

### 2.4 Synthesis of HPQDs via the sharp-edged ultrasonic microreactor

A continuous-flow sharp-edged ultrasonic microreactor system was developed to achieve high-efficiency, controllable synthesis of cesium lead bromide (CsPbBr_3_) HPQDs, leveraging the synergistic effects of sharp-edged microstructure-induced turbulence and ultrasonic cavitation. Two precursor solutions were prepared for synthesis: precursor solution A was formulated by dissolving CsBr (0.2–1.0 mol/L) and PbBr_2_ (0.2–1.0 mol/L, Cs^+^:Pb^2+^ molar ratio = 1:1) in a mixed solvent of OAm, OA, and DMF (volume ratio 1:2:20), stirred at 60 °C for 60 min until fully dissolved; precursor solution B was a mixture of chlorobenzene and castor oil (volume ratio 10:1), used as an anti-solvent to trigger HPQDs nucleation.

A rigorous pre-experiment protocol was implemented to ensure reaction stability: the feed and ultrasonic control modules were activated simultaneously, with pure DMF and chlorobenzene flushed through the microchannels to eliminate contaminants and equilibrate the channel surface. During formal synthesis, Precursor Solutions A and B were continuously injected into the microreactor via micro-syringe pumps at constant flow rates. The flow rate of Precursor Solution B was fixed at 1 mL/min, while that of Precursor Solution A was gradient-adjusted within 100–300 μL/min to investigate the effect of reactant mixing ratio on HPQDs performance. Simultaneously, the effect of precursor concentration was investigated by adjusting the concentrations of CsBr and PbBr_2_ in Precursor Solution A (0.2, 0.4, 0.6, 0.8, and 1.0 mol/L) while maintaining the Cs^+^: Pb^2+^ molar ratio constant.

As the fluid traversed the microchannels integrated with sharp-edged microstructures, an ultrasonic field (21 kHz, 100 W) was applied to induce acoustic cavitation. Under the synergistic action of sharp-edge-induced turbulence and cavitation-derived high-energy effects, intense micromixing and mass transfer intensification were achieved, overcoming diffusion limitations inherent to laminar flow. This high-energy microenvironment triggered instantaneous, synchronized nucleation and controlled crystallization of precursors, enabling continuous production of high-quality HPQDs. Synthesized products were continuously collected at the outlet, stored in a nitrogen-filled vial at 4 °C to preserve optical performance.

### 2.5 Characterization

Numerical simulations were performed using COMSOL Multiphysics 6.2 (COMSOL, Sweden) to design and optimize the structure of the sharp-edged ultrasonic microreactor. The flow field velocity distribution, shear stress, and acoustic pressure gradient within the microchannels were simulated; the average HPQDs concentration across the entire microchannel cross-section was calculated and analyzed to evaluate reaction homogeneity.

X-ray diffraction (XRD, Rigaku D/Max-2500, Cu-Kα radiation, λ = 0.154 nm) and Transmission Electron Microscopy (TEM, JEOL JEM-2100F, accelerating voltage 200 kV) were employed to characterize crystal structure, phase purity, particle size, and morphology at nanoscales. Fourier-transform infrared spectroscopy (FTIR, Thermo Scientific Nicolet iS50) was conducted in the range of 4000–400 cm^−1^ to identify the surface functional groups and chemical bonding configurations. Ultraviolet-visible absorption spectroscopy (UV-Vis Abs, Shimadzu UV-2600), photoluminescence (PL) spectra/photoluminescence excitation (PLE) spectra (Shimadzu RF-6000) were used to determine fundamental optical characteristics, including optical bandgap and emission peak position.

PLQY was measured using an integrating sphere accessory (Hamamatsu C9920-02) coupled to the RF-6000 spectrometer. Photoluminescence decay kinetics were recorded using an FLS980 steady-state/transient fluorescence spectrometer (Edinburgh Instruments, UK) equipped with a time-correlated single photon counting (TCSPC) module (excitation wavelength: 405 nm).

To quantify exciton recombination behavior, experimental fluorescence decay curves were fitted using a multi-exponential decay function based on the non-linear least squares method, mathematically expressed as [38]:

(1) $$A\left(t\right)=\sum_{i=1}^{n}A_{i}\exp\left(-\frac{t}{\tau_{i}}\right)$$

where *A(t)* represents the fluorescence intensity at time t; *τ*_*i*_ and *A*_*i*_ represent the lifetime constant and the corresponding relative amplitude of the decay component, respectively. Based on the fitting parameters, the average carrier lifetime (*τ*_*avg.*_) was calculated using the following equation [39]:

(2) $$\tau_{\mathrm{avg}.}=\frac{A_{1}\tau_{1}^{2}+A_{2}\tau_{2}^{2}}{A_{1}\tau_{1}+A_{2}\tau_{2}}$$

## 3 Results and discussion

### 3.1 Numerical simulation and structural optimization of flow fields in sharp-edged ultrasonic microchannels

To investigate the hydrodynamic behavior and reaction kinetics in sharp-edged ultrasonic microchannels, we established a coupled multiphysics model in COMSOL Multiphysics, integrating the Laminar Flow, Pressure Acoustics, and Chemical Reaction Engineering modules. The core objective was to strengthen vortex formation and intensity within the flow field via geometric optimization of sharp-edged microstructures, thereby boosting HPQDs synthesis concentration and enabling deterministic regulation of the synthetic process.

We simulated flow field characteristics and HPQDs formation behavior across multiple sharp-edged microstructure configurations, with a focus on how key geometric parameters (morphology, height, and inter-structure spacing) modulate fluid dynamics. Quantitative analysis of simulation outputs elucidated the regulatory mechanisms linking these design parameters to vortex intensity and reactant mixing performance. We further established a quantitative, predictive link between flow field topology and the resulting spatial distribution of HPQDs concentration, addressing the longstanding disconnect between microstructural design and deterministic reaction process control. Simulation results confirm that rationally designed sharp-edged microstructures induce strong fluid perturbation and amplify interfacial mass transfer within the microreactor, yielding a highly uniform reactive microenvironment for HPQDs synthesis.

The present simulation assumes isothermal conditions and uses solvent properties at the measured bulk operating temperature. This assumption is reasonable for comparing channel-scale acoustic streaming and mixing trends under controlled bulk-temperature conditions. However, the model does not resolve bubble-scale cavitation collapse, transient local hotspots, or temperature-dependent variations in viscosity and diffusion near cavitation events. A fully coupled non-isothermal acoustic–thermal–cavitation model will be required in future work to quantitatively capture these local thermal effects.

To fluidically validate this active intensification, the flow regimes and cavitation fields were quantitatively characterized. While the pump-driven baseline remains strictly laminar (*R*_*e*_ ≈ 32.99), the 21 kHz acoustic-induced micro-streaming (*U*_*s*_ = 1.2 m·s^−1^) drives the localized Reynolds number (*R_es_*) up to ∼ 949, forcing the flow into a transitional chaotic advection state. Concurrently, the peak negative acoustic pressure reaches *P_neg_* ≈ 2.4 MPa at the structural tips, successfully exceeding the critical solvent cavitation threshold (∼ 1.0–1.5 Mpa at 21 kHz) to initiate violent bubble collapses and localized micro-jets. Consequently, the local Péclet number (*P_e_*) drops by over three orders of magnitude, moving the system out of the diffusion-limited state and dynamically driving the cross-sectional mixing efficiency (*CoV*) from 34.2% to 98.1% within milliseconds.

This highly condensed quantitative boundary firmly substantiates the sonochemical intensification mechanism for uniform HPQDs synthesis. Driven by the initial ultrasonic excitation, the system triggers a collaborative cascade wherein acoustic streaming-dominated micromixing operates in tandem with localized inertial cavitation. This hydrodynamics-to-sonochemistry coupling drastically enhances macro-to-micro mass transfer and achieves rigorous precursor homogenization across the fluidic domain. As a direct consequence of this unified chemical environment, the crystal propagation pathway is redirected toward highly synchronized nucleation and growth kinetics, while concurrently securing optimized surface ligand passivation. Ultimately, this comprehensive structural and chemical refinement directly culminates in the significantly enhanced optoelectronic performance of the resulting HPQDs. Detailed physical properties and step-by-step mathematical derivations supporting this cohesive framework are provided in Supporting Information SI 1, SI 2 and SI 3.

#### 3.1.1 Impact of sharp-edged morphology on the hydrodynamic characteristics of the biomimetic vein-inspired ultrasonic microreactor

**Fig. 2:**
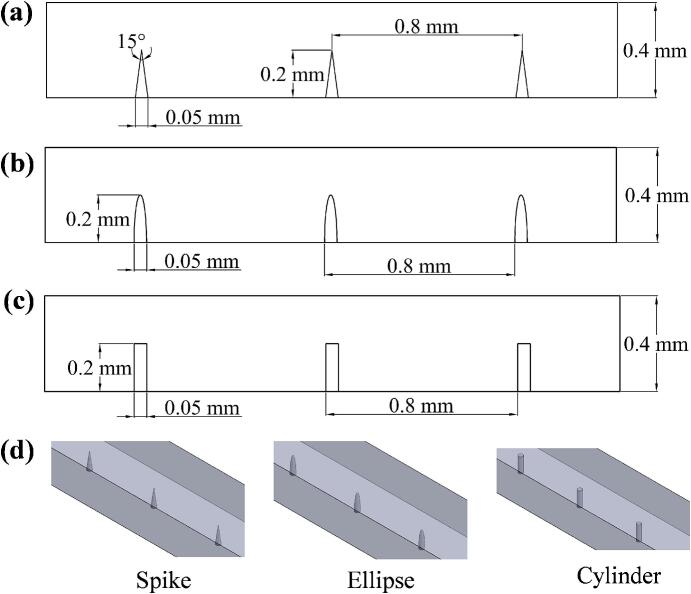
Schematic designs of distinct sharp-edged microstructures for the ultrasonic microreactor: (a) Key structural parameters of the spike microstructure, (b) Key structural parameters of the elliptical microstructure, (c) Key structural parameters of the cylindrical microstructure. (d) Three-dimensional (3D) schematic view of the fabricated sharp-edged microstructures, illustrating their spatial arrangement within the microchannel.

In this subsection, we elucidate how sharp-edged morphology (Fig. 2) modulates hydrodynamic behavior and HPQDs generation efficiency in our biomimetic vein-inspired ultrasonic microreactor. Three distinct microstructural configurations are investigated: spiked, elliptical, and cylindrical geometries, with dimensional parameters detailed in Fig. 2a–c and corresponding 3D models rendered in Fig. 2d. To correlate flow behavior with synthetic performance, we conduct visual and quantitative analysis of HPQDs concentration distributions across both the XOY (Fig. S2a) and ZOX (Fig. S2b) planes of the microchannel, uncovering the intrinsic coupling between flow field topology and mass transfer efficiency.

**Fig. 3:**
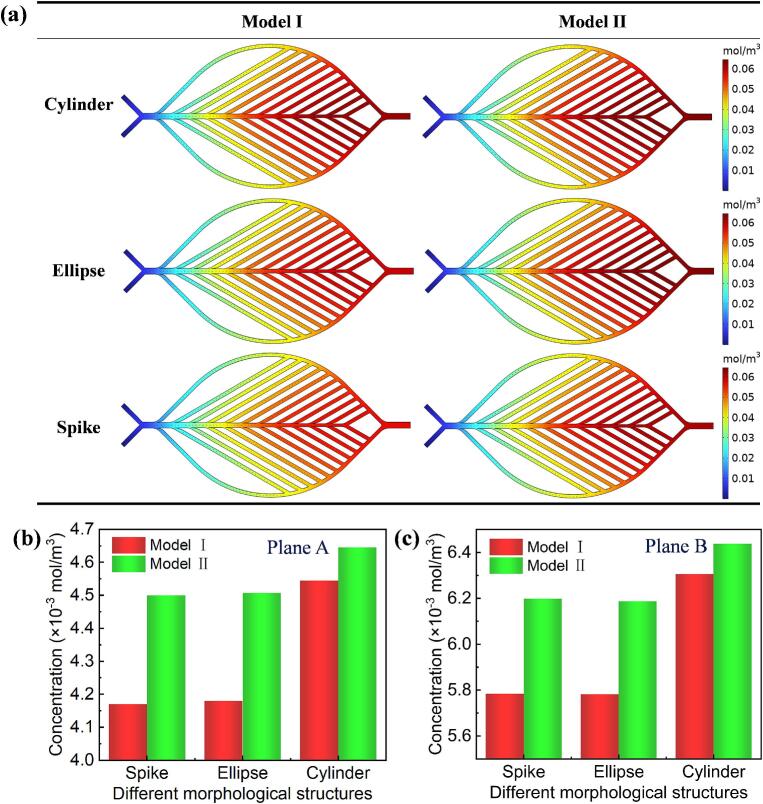
(a) HPQDs concentration distribution maps on the XOY plane for microchannels with different sharp-edged morphologies. (b) Average HPQDs concentrations on the ZOX plane (Plane A), and (c) average HPQDs concentrations at the outlet cross-section (Plane B), corresponding to the microchannels with different sharp-edged morphologies.

We first elucidate the synergistic regulatory mechanism of the ultrasonic field on fluid dynamics and mass transfer within the microreactor, using HPQDs concentration contours in the XOY plane (Fig. 3a) and pressure field distributions (Table S2) as the core quantitative basis. In the ultrasound-off control (Model I), the fluid exhibits canonical laminar flow characteristics, with a sharp, well-defined diffusion boundary between precursor streams. Pressure decays rapidly along the flow direction, yielding insufficient driving force to promote fluid penetration into the leeward recirculation zones of the microstructures. This ultimately forms extensive low-concentration mass transfer dead zones at the channel sidewalls — a well-documented intrinsic limitation of conventional laminar microfluidic systems.

In stark contrast, applying the ultrasonic field (Model II) induces a fundamental reconfiguration of the hydrodynamic environment. For the pressure field, ultrasonic cavitation generates transient shock waves, high-velocity microjets, and localized pressure fluctuations, which markedly expand the coverage of high-pressure regions and optimize the spatial distribution of the pressure gradient (Table S2). The amplified pressure driving force effectively overcomes microchannel flow resistance, forcing the fluid to break through the confinement of the viscous laminar boundary layer. This eliminates stepwise concentration gradients, blurs the physical interfaces between discrete concentration zones, and drives a critical transition from diffusion-dominated to convection-dominated mass transfer — directly resolving the longstanding diffusion limitation bottleneck of laminar microchannel flows.

Beyond the in-plane (XOY) synergistic modulation detailed above, analysis of the ZOX plane reveals the critical role of our microstructures in mitigating the intrinsic vertical stratification effect ubiquitous in laminar microfluidic systems. As shown in the ZOX plane concentration contours (Fig. S3), the sharp-edged microstructures act as hydrodynamic modulators: under ultrasonic excitation, they generate coherent 3D vortex structures that drive intensive convective mixing along the channel height (Z-axis) — a prerequisite for spatially uniform HPQDs synthesis across the full 3D reactor volume. Under Model II, the cylindrical microstructure delivers the most homogeneous Z-direction concentration distribution, with full coverage of high-concentration reaction zones across the entire channel height. This confirms complete elimination of vertical concentration gradients, enabling synchronous nucleation and uniform growth of HPQDs nanocrystals — a critical requirement for synthesizing high-quality HPQDs with narrow FWHM.

To quantitatively benchmark the impact of sharp-edged morphology on HPQDs synthesis efficiency, we statistically analyzed the volume-averaged HPQDs concentration across the ZOX plane (Fig. 3b) and microreactor outlet cross-section (Fig. 3c). Relative to the Model I, Model II yields a universal increase in average HPQDs concentration, validating the broad applicability of ultrasonic field assistance for boosting reaction conversion in microfluidic synthesis. Of the three microstructural geometries, the cylindrical configuration delivers superior synthetic performance: under ultrasonic excitation, the outlet cross-sectional HPQDs concentration reaches a maximum of 6.44 × 10^−3^ mol/m^3^, markedly outperforming the spiked (6.20 × 10^−3^ mol/m^3^) and elliptical (6.19 × 10^−3^ mol/m^3^) counterparts. This quantitative analysis definitively demonstrates the inherent advantage of the cylindrical microstructure in HPQDs production efficiency, identifying it as the optimal geometric design for high-yield, precisely controllable HPQDs synthesis.

Microscopic analysis of flow streamlines and velocity vectors (Table 1) elucidates the hydrodynamic origins of the observed morphology-dependent synthesis performance, establishing a direct, quantitative link between geometric design and HPQDs synthetic outcomes. For the spiked microstructure, while its sharp-tip geometry is thermodynamically favorable for cavitation nucleation, its inherent geometric discontinuities trigger abrupt flow constriction and disordered vortex shedding. Under high-intensity ultrasonic excitation, these localized vortices are excessively amplified, degenerating the flow field into unregulated, highly chaotic flow that disrupts the synchronous HPQDs nucleation required for uniform nanocrystal growth. For the elliptical microstructure, its continuously varying curved profile induces strong spatial fluctuations in the velocity gradient, inhibiting stable attachment of the laminar boundary layer. This results in intermittent, unstable mass transfer that degrades the homogeneity of the reactive microenvironment critical for high-quality HPQDs synthesis.

**Table 1:** Local streamline diagrams of microchannels integrated with distinct sharp-edged morphologies (spiked, elliptical, and cylindrical), illustrating the hydrodynamic characteristics associated with each configuration under ultrasonic excitation.

In stark contrast, the cylindrical microstructure exhibits exceptional hydrodynamic performance: its symmetric, smooth geometric profile maintains parallel, ordered streamlines downstream, inherently suppressing excessive localized turbulence. This design achieves an optimal balance between two competing core performance metrics: (1) leveraging ultrasonic microjets to disrupt the laminar boundary layer and intensify mass transfer, and (2) preserving global flow order to ensure consistent, predictable reaction kinetics. The resulting regulated turbulence — turbulent mixing confined within a globally ordered flow framework — enables precise synergy between mass transfer enhancement and flow stability. Collectively, these inherent hydrodynamic advantages underpin the superior HPQDs production efficiency of the cylindrical microstructure, validating it as the optimal geometric design for high-yield, highly controllable HPQDs synthesis.

#### 3.1.2 Effect of microstructure height on hydrodynamic performance of the biomimetic vein-inspired ultrasonic microreactor

To elucidate the impact of microstructure height (*H*) on hydrodynamic behavior and HPQDs synthesis efficiency, we simulated five sets of sharp-edged microstructures with gradient heights (*H* = 0.1–0.3 mm, Fig. S4a), and systematically compared their mass transfer characteristics under two modes: Model I and Model II. Simulation results show that HPQDs concentration field evolution exhibits strong height-dependent behavior, governed by the coupling efficiency between microstructure geometry and the ultrasonic field. Under Model I, fluid flow is confined by the laminar boundary layer, with mass transfer dominated by inefficient molecular diffusion, resulting in localized high-concentration regions and highly heterogeneous gradient distributions. In contrast, Model II triggers significant mass transfer intensification via ultrasonic cavitation; notably, this enhancement is non-linear, as varying microstructure heights yield distinct acoustic energy coupling efficiencies (driven by height-dependent acoustic pressure amplification), leading to differentiated HPQDs concentration field reconstruction and mass transfer performance.

Cross-comparison across microstructural morphologies further reveals their differential responses to height variation. For the spiked microstructure (Table S3), HPQDs concentration uniformity is fundamentally limited by its geometric discontinuities: even though the reaction zone expands to 60% of the channel under Model II, severe flow separation induces discontinuous concentration gradients, precluding homogeneous spatial distribution. For the elliptical microstructure (Table S4), streamlines remain smooth at low heights; however, increasing height (especially *H* = 0.3 mm) renders the curved flow channels prone to localized concentration fluctuations and steep gradient shifts under strong acoustic perturbation, degrading reaction stability.

In stark contrast, the cylindrical microstructure (Table 2) exhibits exceptional gradient homogenization capability across the XOY plane. Unlike Model I, where marked concentration discrepancies exist between main and branch channels, Model II delivers spatially uniform filling of high-concentration regions across the entire channel. This performance arises from the cylindrical microstructure’s efficient acoustic energy coupling, which translates localized reaction intensification into global, homogeneous synthesis — directly overcoming the bottleneck of inadequate local reaction efficiency inherent to Model I. Height optimization further amplifies this advantage: moderate heights (*H* = 0.2–0.3 mm) balance acoustic pressure amplification and flow field stability, maximizing both HPQDs concentration and spatial uniformity (evident in ZOX plane distributions, Table 2).

**Table 2:** HPQDs concentration distribution maps on the XOY and ZOX planes of cylindrical microchannels with different sharp-edged heights, under both Model I and Model II modes.

**Fig. 4:**
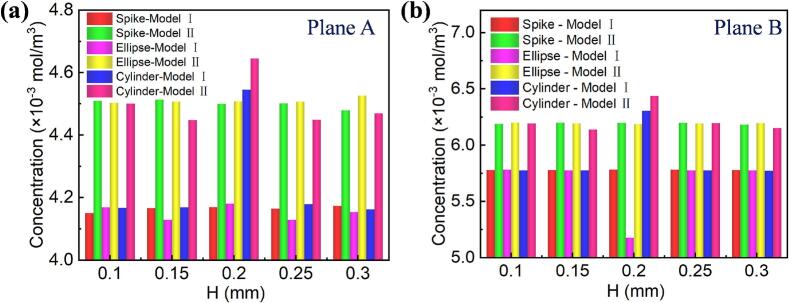
(a) Average HPQDs concentrations on the ZOX plane (Plane A) for microchannels with different sharp-edged heights, under the two physical field modes. (b) Average HPQDs concentrations at the outlet cross-section (Plane B) for microchannels with different sharp-edged heights, comparing Model I and Model II modes.

Further quantitative analysis of height-dependent HPQDs synthesis performance (Fig. 4) reveals a non-monotonic relationship between microstructure height and hydrodynamic efficiency, governed by the trade-off between acoustic energy coupling and flow resistance. In the low-height regime (*H* = 0.1–0.15 mm), microstructure-induced fluid perturbation is weak, leading to negligible performance differences between Model I and Model II and limited mixing enhancement. This is attributed to insufficient interaction between the low-aspect-ratio microstructures and the ultrasonic field, which fails to effectively disrupt the laminar boundary layer or intensify convective mass transfer. In the high-height regime (*H* = 0.25–0.3 mm), excessive flow resistance induces prominent stagnant wake zones (mass transfer dead zones), compromising the spatial homogeneity of the reactive microenvironment. Critically, only at the optimal height of *H* = 0.2 mm does the system achieve the perfect balance between acoustic energy coupling, fluid perturbation intensity, and flow resistance. Quantitative data confirm that at this height, the cylindrical microstructure delivers the maximum performance enhancement from Model I to Model II, with peak average concentrations at Plane A and Plane B reaching 4.65 × 10^−3^ mol/m^3^ and 6.44 × 10^−3^ mol/m^3^, respectively. This outperforms all other height configurations, validating the critical role of height optimization in maximizing HPQDs synthesis efficiency and uniformity.

To decode the mechanism by which sharp-edged microstructure height governs mass transfer efficiency, we performed high-resolution visualization and analysis of streamline field characteristics across all configurations. Systematic analysis of flow field evolution as a function of height reveals that sharp-edged morphology exerts a defining influence on flow stability, with fundamentally distinct hydrodynamic responses across the three microstructural geometries. For the spiked microstructure (Table S5), hydrodynamic behavior is inherently constrained by its geometric discontinuities. Regardless of height increase, its sharp windward edge induces abrupt changes in incoming fluid momentum, triggering severe flow separation on the leeward side. This separation persists even at the optimal height, generating stagnant dead zones that disrupt homogeneous mass transfer — consistent with the limited HPQDs concentration enhancement observed in Fig. 4.

For the elliptical microstructure (Table S6), hydrodynamic performance is highly sensitive to height variation. At low heights, streamlines remain smoothly attached to the curved profile, enabling relatively stable mass transfer. However, increasing height to *H* = 0.3 mm amplifies pressure gradient fluctuations within the boundary layer (driven by the extended curved surface), readily triggering curvature-induced flow instability and parasitic secondary flow oscillations. These unregulated oscillations disrupt global flow order, leading to spatiotemporal fluctuations in mass transfer that degrade HPQDs concentration and size uniformity.

In stark contrast, the cylindrical microstructure (Table 3) exhibits exceptional geometric and hydrodynamic robustness. Critically, at the optimal height of *H* = 0.2 mm, this configuration achieves an ideal hydrodynamic equilibrium: it fully circumvents the stagnant dead zones of spiked structures and the oscillatory instability of elliptical structures, delivering a uniform, highly ordered, periodic wavy streamline distribution across the entire flow field. This flow pattern reflects the cylindrical geometry’s unique ability to balance mixing intensification and hydraulic flow resistance, driving a transition from canonical laminar flow to a state of quasi-ordered regulated turbulence (consistent with the core mechanism established in our preceding analysis). This transition maximizes mass transfer efficiency (evident in the peak HPQDs concentrations in Fig. 4) while preserving global flow stability. These results further corroborate that the cylindrical microstructure at *H* = 0.2 mm delivers superior hydrodynamic performance in our biomimetic vein-inspired ultrasonic microreactor, directly translating to enhanced HPQDs synthesis efficiency and uniformity — definitively validating it as the optimal structural configuration.

**Table 3:** Local streamline diagrams of cylindrical microchannels at different sharp-edged heights, illustrating flow field characteristics under Model I and Model II modes.

#### 3.1.3 Effect of inter-cylinder spacing on hydrodynamic performance of the biomimetic vein-inspired ultrasonic microreactor

**Fig. 5:**
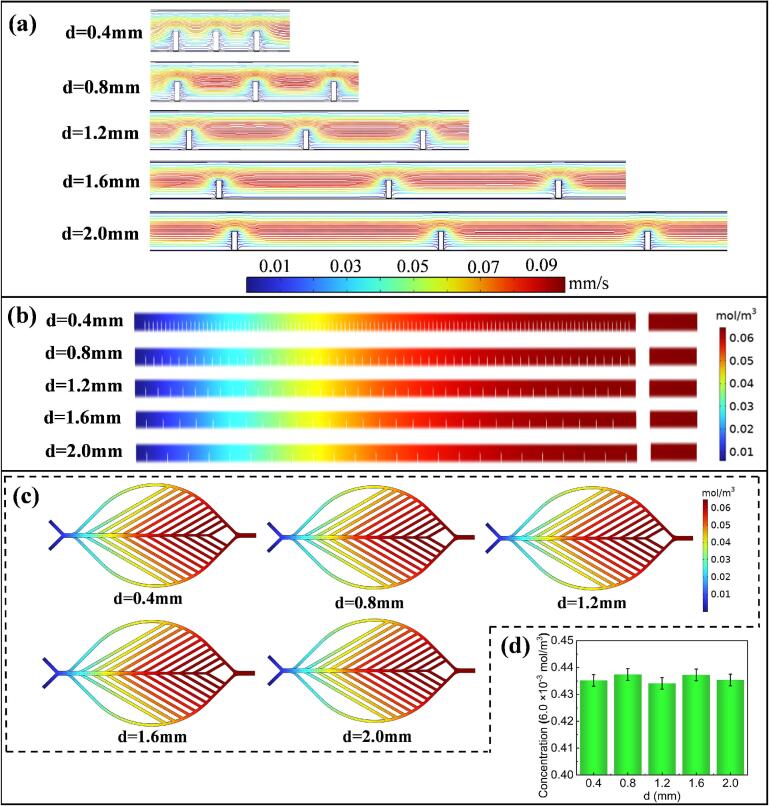
(a) Local streamline diagrams of cylindrical microchannels with different inter-structure spacing, under ultrasound-on (Model II) mode. (b) HPQDs concentration distribution maps on the ZOX plane. (c) HPQDs concentration distribution maps on the XOY plane. (d) Average HPQDs concentrations at the outlet cross-section (Plane A), for cylindrical microchannels with varying inter-structure spacing and fixed optimal height (*H* = 0.2 mm).

Building on the optimized cylindrical microstructure and critical height identified in the preceding sections, we investigate five sets of microstructures with gradient inter-cylinder spacing (*d*) (Fig. S4b), to decode how array density modulates hydrodynamic behavior and HPQDs synthesis efficiency — a critical prerequisite for scalable, uniform microfluidic synthesis. Analysis of streamline evolution within the microchannels (Fig. 5a) reveals that spacing exerts a defining regulatory role in the continuity of flow perturbation, with flow field morphology exhibiting a strong non-monotonic dependence on inter-cylinder spacing.

In the small-spacing regime (*d* = 0.4 mm), excessive geometric constriction of the flow channel induces a pronounced blockage effect, which inhibits the full development and shedding of cavitation-induced vortices. This results in severely insufficient mixing kinetics and elevated hydraulic flow resistance relative to the critical spacing, ultimately limiting reactant mixing and HPQDs nucleation. Conversely, in the large-spacing regime (*d* ≥ 1.2 mm), the sparse array arrangement allows perturbations generated by upstream cylinders to fully dissipate before reaching downstream structures. This causes clear flow re-laminarization, breaking the perturbation transmission chain and reverting to the diffusion-limited mass transfer inherent to conventional smooth-walled microchannels. Only at the critical spacing of *d* = 0.8 mm does the flow field enter an optimal wake synergistic interference regime — a core feature of high-efficiency array design. Here, wake vortices shed from upstream cylinders continuously and effectively impinge on downstream cylinders, generating a coherent, fluctuating, high-intensity perturbation streamline network across the entire channel. This coherent vortex chain mechanism maximizes convective mixing efficiency while avoiding ineffective momentum dissipation, directly overcoming the limitations of both overly dense and sparse array configurations.

Further analysis of HPQDs concentration distribution evolution across the ZOX (Fig. 5b) and XOY (Fig. 5c) planes quantitatively demonstrates the reaction kinetic differences across spacing configurations, with concentration patterns highly sensitive to cylindrical array density. The *d* = 0.8 mm configuration delivers an optimal global filling effect: the channel exhibits the most saturated, continuous high-concentration regions, with high-concentration products fully filling the main vein and smoothly penetrating the terminal branch veins at the channel edges. The entire biomimetic vein network shows homogeneous concentration distribution, confirming that reactants maintain an ideal residence time distribution (RTD) within the complex bifurcated structure.

In contrast, the channel with *d* = 0.4 mm (overly dense array) shows overall low concentration, as elevated flow resistance inhibits effective mass transfer and induces localized stagnant zones. For the sparse array (e.g., *d* = 2.0 mm), although localized reactions are triggered, discontinuous flow perturbations lead to significant concentration attenuation and discontinuities across the global distribution, with steep concentration gradient drops observed at vein terminals and secondary branches. These phenomena collectively demonstrate that mismatched flow resistance (overly dense arrays) or discontinuous mixing (sparse arrays) induces localized reaction insufficiency, precluding high-quality, uniform HPQDs synthesis across the entire biomimetic vein network. These results confirm *d* = 0.8 mm as the optimal inter-cylinder spacing, validating the synergistic balance between array density, flow field stability, and mass transfer efficiency.

To quantify the impact of inter-cylinder spacing on HPQDs synthesis efficiency, we analyzed the volume-averaged HPQDs concentration at the microreactor outlet cross-section (Fig. 5d). Our results reveal a non-monotonic relationship between average outlet concentration and inter-cylinder spacing, rather than random fluctuations. Specifically, at the critical spacing of *d* = 0.8 mm, the average HPQDs concentration reaches a peak of 6.44 × 10^−3^ mol/m^3^, significantly outperforming both dense and sparse array groups. While the average concentration at *d* = 1.6 mm remains relatively high, this configuration compromises the core miniaturization and space utilization requirements of microfluidic devices. In contrast, *d* = 0.8 mm achieves an optimal trade-off between space utilization and mass transfer efficiency, while maintaining superior flow field continuity and homogeneous global HPQDs distribution — critical advantages for scalable microfluidic synthesis.

Therefore, systematic simulation analysis and multi-parameter optimization of sharp-edged microstructure morphology, height, and spacing enable us to identify the optimal configuration for the biomimetic vein-inspired ultrasonic microreactor: a cylindrical microstructure array with height *H* = 0.2 mm and inter-structure spacing *d* = 0.8 mm. Mechanistically, this optimized microstructure geometry dramatically amplifies local acoustic pressure gradients and acoustic field localization. This spatial confinement potentially heightens cavitation sensitivity and intensifies acoustic streaming-induced micromixing, rather than serving as direct evidence for a reduction in the intrinsic cavitation threshold. This optimized parameter combination constructs an ideal reactive microenvironment that synergizes high mixing efficiency and global flow field stability. Importantly, this configuration establishes a deterministic quantitative link between mechanical microstructure design and nanomaterial synthesis performance, laying a robust engineering foundation for the subsequent controllable, high-yield synthesis of high-performance HPQDs.

### 3.2 Tuning of HPQDs properties via the optimized sharp-edged ultrasonic microreactor

**Fig. 6:**
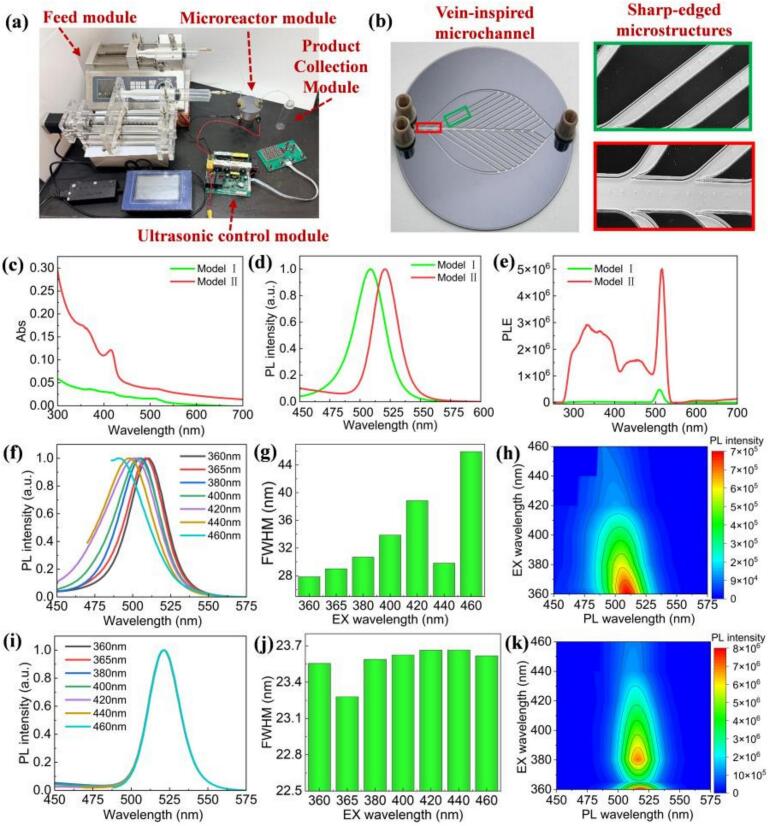
Characterization of the sharp-edged ultrasonic microreactor platform and optoelectronic properties of as-synthesized HPQDs. (a) Custom-built sharp-edged ultrasonic microreactor system platform. (b) biomimetic vein-inspired microchannel integrated with sharp-edged microstructures. (c–e) Comparative optical characterization of HPQDs synthesized under Model I and Model II: (c) UV–Vis absorption spectra, (d) steady-state PL emission spectra, (e) PLE spectra. (f–h) Detailed PL characterization of Model I HPQDs: (f) 2D normalized PL spectra, (g) FWHM, (h) 3D PL spectra under excitation wavelengths of 360–460 nm. (i–k) Corresponding PL characterization of Model II HPQDs under identical excitation conditions: (i) 2D normalized PL spectra, (j) FWHM, (k) 3D PL spectra.

Building on the geometric optimization and mechanistic framework from Section 3.1, we performed systematic experimental validation using HPQDs synthesis as a model reaction. Using the optimized sharp-edged ultrasonic microreactor platform (Fig. 6a-b), we investigated the effects of key operating parameters on HPQDs morphological and optoelectronic properties. This work enables precision-controlled synthesis of HPQDs with high color purity and exceptional PLQY, establishing a framework to bridge lab-scale sonochemical synthesis and industrial translation.

#### 3.2.1 Validation of ultrasonic synthesis enhancement

To quantify the effect of ultrasonic excitation on HPQDs synthesis, we performed a comparative study using the optimized cylindrical microstructure array (*H* = 0.2 mm, *d* = 0.8 mm) under Model I (ultrasound-off control, 0 kHz) and Model II (ultrasonic excitation, 21 kHz/100 W). All experiments were performed at room temperature with fixed parameters: 150 μL/min flow rate for 0.4 mol/L Precursor A, 1 mL/min for Precursor B, ensuring identical reaction condition.

**Fig. 7:**
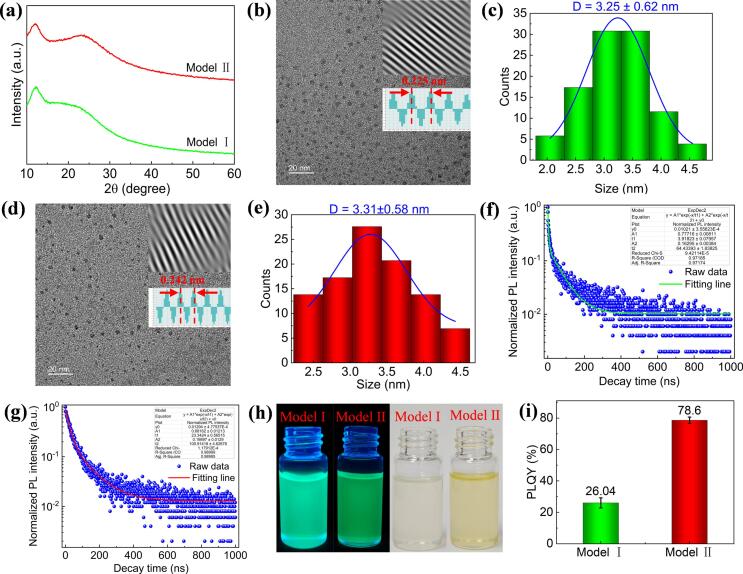
Structural, morphological, and photophysical characterization of Model I and Model II HPQDs. (a) XRD patterns; (b–c) TEM image (b) and particle size distribution (c) of Model I HPQDs; (d–e) TEM image (d) and particle size distribution (e) of Model II HPQDs; (f–g) TRPL decay curves of Model I (f) and Model II (g) HPQDs; (h) HPQDs solution photographs under natural light and UV excitation; (i) Absolute PLQY values.

Comprehensive characterization of HPQDs optoelectronic properties, crystal structure, and morphology (Fig. 6, Fig. 7) reveals that ultrasonic cavitation dominates synthetic performance, consistent with Section 3.1 simulation results. UV-vis absorption spectra (Fig. 6c) show weak absorption for Model I, consistent with diffusion-limited mixing and low HPQDs yield, while Model II exhibits markedly enhanced broadband absorption with a well-defined exciton peak at ∼ 415 nm. This difference arises from cavitation-generated localized high temperatures, pressures, and high-velocity microjets, which intensify precursor mixing and mass transfer, driving an order-of-magnitude yield increase. PL emission spectra (Fig. 6d) show a red shift of the emission maximum from 509 nm (Model I) to 521 nm (Model II). Based on the quantum confinement effect, this bathochromic shift demonstrates that the ultrasonic field modulates HPQDs growth kinetics, promoting evolution to a thermodynamically stable mature stage and yielding HPQDs with enhanced crystallinity. PLE spectra (Fig. 6e) further corroborate this enhancement, with Model II showing orders-of-magnitude higher excitation intensity than Model I, indicating that ultrasonic excitation passivates HPQDs surface trap states and suppresses non-radiative recombination, the key determinants of photoluminescence efficiency.

Excitation wavelength-dependent PL analysis was performed to quantify HPQDs size uniformity and electronic energy level structure. As shown in Fig. 6f, Model I HPQDs exhibit pronounced excitation-dependent emission behavior: as the excitation wavelength increases from 360 nm to 460 nm, the PL emission peak undergoes a significant red shift, accompanied by large, irregular fluctuations in FWHM (Fig. 6g). This is a characteristic hallmark of polydisperse nanocrystal systems, arising from abundant surface trap states, where HPQDs of varying sizes or defect levels are selectively excited by photons of different energies — consistent with the diffusion-limited, inefficient mixing predicted and observed for Model I in preceding sections.

In stark contrast, Model II HPQDs show near-ideal excitation wavelength independence (Fig. 6i): their emission peak remains stable at ∼ 521 nm across all tested excitation wavelengths, with FWHM consistently confined to an ultra-narrow range of 23.0–23.7 nm (Fig. 6j). This result definitively demonstrates that the shear forces generated by ultrasonic cavitation homogenize the supersaturation degree across the entire reaction zone, enabling synchronous nucleation and uniform growth of all crystal nuclei — yielding HPQDs with exceptional monodispersity and color purity. The optimized ultrasonic microreactor produced HPQDs with a narrow FWHM of 23.28 nm. Although this value is not an absolute record among CsPbBr_3_ and related HPQDs, it is comparable to the linewidths reported for high-quality HPQDs prepared by mainstream synthesis strategies. More importantly, this narrow linewidth is achieved under continuous-flow and high-throughput conditions, indicating that the ultrasonic microreactor can preserve optical spectral quality while improving process scalability.

Quantitatively, this performance leap is rigorously corroborated by the 3D PL matrices. Model I exhibits a suppressed peak intensity of merely 7.0 × 10^5^ a.u. (Fig. 6h), characterized by a weak, diffuse, and oblate energy contour that undergoes a severe excitation-dependent shift. This profile quantitatively proves the coexistence of significant size polydispersity and dense surface defect states that trigger dominant non-radiative exciton recombination. In sharp contrast, the peak intensity of Model II skyrockets to 8.0 × 10^6^ a.u. (Fig. 6k) — representing a substantial 11-fold quantitative enhancement—while presenting a highly localized and symmetric “bullseye” spectral contour. This fundamental spectral transformation underscores the absolute dominance of intrinsic exciton recombination, confirming that the high-energy physical field effects of ultrasonic cavitation effectively optimize surface ligand coordination and minimize dangling bonds as the core determinants of the enhanced optoelectronic performance.

Crystal structure and surface morphology characterization further elucidate the physical origins of the observed optoelectronic enhancement, establishing a direct link between ultrasonic-induced mechanical effects (acoustic streaming, cavitation) and HPQDs structural quality. XRD patterns (Fig. 7a) reveal that Model I exhibit a nearly featureless diffraction profile in the 20°–30° range, indicating the absence of long-range periodic lattice formation under conventional laminar flow conditions. With the application of the ultrasonic field, Model II develops a distinct broad diffraction halo centered at 21°–25°, which matches the primary diffraction planes of the standard cubic CsPbBr_3_ perovskite phase (PDF card #18-0364) [40], [41], [42]. This evolution fundamentally demonstrates that the extreme localized energy from cavitation hotspots successfully overcomes the perovskite crystallization activation barrier, driving short-range ordering of the perovskite lattice. It's worth noting that these broad diffraction features must be interpreted with caution. Rather than indicating poor crystalline quality, the pronounced broadening of the diffraction peaks is highly characteristic of quantum dots with ultrasmall dimensions, where the finite crystal size and limited coherent diffraction length inherently govern the profile according to the Scherrer relationship. Furthermore, the dense coverage of organic surface ligands, the intrinsically low scattering volume of the nanoscale crystallites, and possible local microstrain concurrently weaken and broaden the diffraction signals. Therefore, while these XRD insights demonstrate the successful emergence of nanoscale crystalline CsPbBr_3_ domains under intense cavitation-induced shear forces and rapid reaction quenching, Fig. 7a alone does not constitute definitive proof of complete phase purity or macroscopic crystallinity, thus requiring further corroboration by high-resolution local structural characterization.

TEM microscopic observations directly manifest the definitive role of the acoustic field in shaping the morphology of the synthesized HPQDs (Fig. 7b–e). Due to the inherent thermodynamic stability of the cubic phase, the nanocrystals consistently maintain a well-defined cubic profile across all examined operating conditions, exhibiting no systematic shape transitions (e.g., spherical-to-cubic evolution) (Fig. S5). Under the optimized acoustic regime, the synergistic coupling of acoustic streaming shear forces and localized ultrasonic cavitation promotes highly uniform mechanical energy dissipation and synchronized ligand capping kinetics across all crystalline facets. This active sonochemical stabilization effectively suppresses ultrasound-induced ligand shedding, fragmentation, or crystal aggregation, thereby substantially advancing the morphological uniformity. Statistically, this tailored microenvironment not only leads to a modest shift in the average particle size from 3.25 ± 0.62 nm (Model I) to 3.31 ± 0.58 nm (Model II) within the quantum-confined regime, but also constricts the coefficient of variation (*CV*) of the size distribution from 19.1% to 17.5%, verifying a significantly improved size monodispersity.

Delving into the atomic-scale structure, ultrasonic cavitation-induced energetic effects effectively modulate the internal atomic arrangement of the nanocrystals. Specifically, the lattice fringe spacing undergoes an optimization from 0.225 nm to 0.242 nm, aligning precisely with the standard (211) crystalline plane of cubic CsPbBr_3_. This structural lattice relaxation directly underscores the successful elimination of internal crystal defects, such as lattice distortions and vacancies. Furthermore, within the resolution and sampling boundaries of the HRTEM/FFT analysis, no auxiliary lattice d-spacings indexable to common impurity phases (e.g., PbBr_2_ or CsBr) were detected. While this localized characterization stops short of claiming absolute macroscopic phase purity, it firmly demonstrates that crystalline CsPbBr_3_ is the exclusively predominant phase formed within the investigated domains, corroborating the superior crystalline and structural refinement enabled by the microreactor design.

Recent studies have shown that the optoelectronic properties of HPQDs are closely correlated with their morphology, quantum-confinement characteristics, surface chemistry, and lattice ordering [43], [44]. In our ultrasonic microreactor, acoustic-field-enhanced micromixing and mass transfer promote more homogeneous nucleation and growth, yielding HPQDs with improved morphological uniformity and reduced lattice distortion. Such structural regulation can narrow the energetic disorder within the quantum-confined ensemble, thereby suppressing inhomogeneous broadening of the emission linewidth. Meanwhile, improved surface passivation and local crystallographic ordering reduce trap-assisted non-radiative recombination pathways, contributing to the enhanced PLQY, narrowed FWHM, and prolonged carrier lifetime of the optimized HPQDs.

FTIR spectroscopy was performed to elucidate the chemical composition and to evaluate the sonochemical promotion effect on the synthesis of HPQDs by comparing Model I and Model II, with results shown in Fig. S6. Both spectra exhibit characteristic absorption bands that confirm the successful construction of the HPQDs framework. Specifically, the sharp peak at ∼2925 cm^−1^ is assigned to the C-H/O-H stretching vibrations, while the distinct peak observed at ∼2360 cm^−1^ is attributed to the stretching vibration of C≡N groups, representing the degree of nitrogen doping. Additional characteristic peaks are assigned to the C-O stretching mode (∼1265 cm^−1^), C-H bending (∼1035 cm^−1^), and the C-Cl/C-H vibrations (∼ 798 cm^−1^). Notably, Model II exhibits enhanced absorption intensities for these functional groups compared to Model I, providing direct evidence of the sonochemical promotion effect. The intense acoustic cavitation and resulting localized “hotspots” effectively lower the activation energy for bond cleavage and radical recombination, facilitating a higher density of surface functional group grafting and superior structural stability.

TRPL spectroscopy reveals that the *τ*_*avg.*_ of HPQDs increases significantly from 50.59 ns (Model I) to 65.78 ns (Model II) under ultrasonic excitation (Table S7). This marked enhancement uncovers the sonochemical regulatory mechanism of exciton dynamics in HPQDs. For Model I (Fig. 7f), the fast decay lifetime τ_1_, corresponding to non-radiative recombination via surface trap states, is only 3.92 ns with a high amplitude fraction of 78%, reflecting the high density of unpassivated trap states in the control sample. For Model II (Fig. 7g), τ_1_ increases dramatically to 23.34 ns (a nearly 6-fold increase), definitively demonstrating that the in situ annealing effect from cavitation-generated transient high energy effectively repairs surface dangling bonds and vacancy defects. Meanwhile, the slow decay component τ_2_, corresponding to intrinsic radiative recombination, increases from 64.43 ns to 100.91 ns, confirming that ultrasound-induced lattice reconstruction improves crystal integrity and reduces phonon scattering from lattice disorder. This simultaneous suppression of non-radiative recombination channels and extension of radiative recombination lifetime directly corroborates the lattice optimization observed via TEM at the exciton dynamics level, revealing the fundamental mechanism by which sonochemical methods achieve a qualitative leap in HPQDs performance via precise energy injection, without altering the material’s chemical composition and ligand environment (as confirmed by FTIR spectroscopy).

The profound suppression of non-radiative recombination directly translates into a remarkable enhancement in absolute photoluminescence quantum yield (PLQY). Specifically, Model II HPQDs achieve an impressive peak PLQY of 78.6% (Fig. 7i), representing an approximate three-fold increase over the 26.04% observed for Model I. This substantial performance leap is underpinned by exceptional macro-to-micro replication fidelity (n ≥ 3): Model II strictly constricts batch-to-batch PLQY variance to a tight cluster (RSD = 2.6%), in stark contrast to the stochastic scattering of Model I (26.04% ± 3.2%, RSD = 12.3%). Concurrently, the micro-spectral linewidth (full width at half maximum, FWHM) is tightly locked within a narrow, uniform envelope (23.0–23.7 nm) across a broad excitation spectrum (360–460 nm, Table S8), effectively suppressing structural polydispersity.

**Fig. 8:**
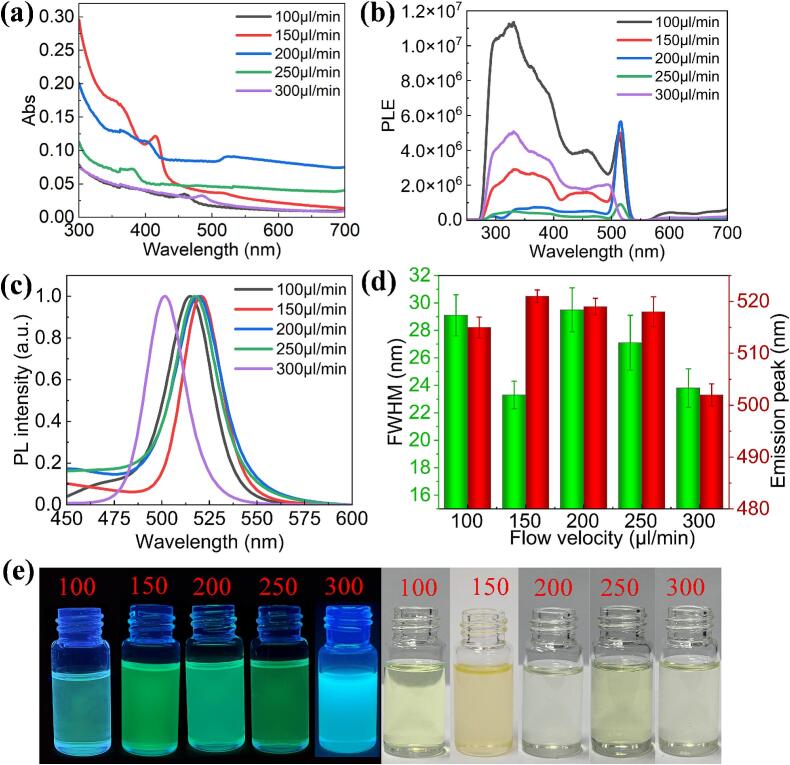
Flow rate-dependent optoelectronic property tuning of HPQDs in the optimized sharp-edged ultrasonic microreactor: (a) UV–vis absorption spectra, (b) PLE spectra, (c) steady-state PL emission spectra, (d) quantitative statistics of FWHM and emission peak positions across all flow rates, (e) optical fluorescence photographs of HPQDs solutions under 365 nm UV and sunlight irradiation.

This stringent structural control fundamentally originates from the optimized hydrodynamic and sonochemical environments within the microreactor. Multi-physics simulations predict that under targeted ultrasonic irradiation, the pronounced intensification of acoustic boundary layer perturbations drastically enhances the spatial uniformity of the local concentration field. This computational projection is phenomenologically validated by the experimental flow-rate-dependent FWHM evolution (Fig. 8d), wherein the emission linewidth reaches an absolute minimum of 23.28 nm at an optimized flow rate of 150 μL/min, directly demonstrating a radical mitigation of mass-transfer-induced system inhomogeneity. Moreover, the robust operational stability of this acoustically driven reaction environment is corroborated by extended reproducibility datasets in Table S8, which reveal that the FWHM RSD remains consistently below 2.6% within the primary operating windows. Crucially, while these collective optical and statistical metrics offer compelling indirect functional evidence of the enhanced micro-mixing trends, they do not constitute a direct, vis-à-vis verification of the local fluidic vortex topologies. To address this inherent characterization limit, advanced real-time optical diagnostics, such as micro-particle image velocimetry (μ-PIV) or laser-induced fluorescence (LIF) dye-mixing experiments, are warranted in future investigations to achieve a direct, quantitative validation of the simulated velocity and concentration fields.Optical photographs further validate this performance leap (Fig. 7h): under natural light, Model II HPQDs exhibit a saturated yellow-green color, while under 365 nm UV excitation, they emit bright, uniform deep-green fluorescence with no visible quenching. In contrast, Model I HPQDs appear pale yellow under natural light and show weak, heterogeneous fluorescence under UV excitation. Collectively, these structural, optical, and dynamic characterizations unequivocally confirm the decisive role of the ultrasonic field, synergized with the optimized sharp-edged microstructures, in enabling the controllable synthesis of high-quality, high-efficiency HPQDs.

To evaluate the industrial potential and process reliability of the proposed method, comparative studies were performed without ultrasound (Model I) and with ultrasound (Model II) (Fig. S7). As shown in Fig. S7c, introducing the ultrasonic field in Model II substantially increased the production yield from 42.3% to 73.8%, indicating superior scalability with minimized material waste. Furthermore, operational robustness was verified under distinct environmental conditions. In the photostability test under a 365 nm UV lamp (Fig. S7a), Model II exhibited a significantly stabilized normalized PL intensity trajectory compared to Model I. Crucially, to simulate practical storage environments, a long-term stability test was conducted under continuous ambient light exposure (Fig. S7b). Model II displayed a remarkably flattened degradation slope over 50 hours, maintaining a high PLQY retention rate of 75%, markedly outperforming the 65% retention of Model I (Fig. S7d). These results confirm that the optimized ultrasonic process delivers the high reproducibility, scalability, and robust storage stability required for commercial applications.

To further clarify the advantages of the optimized ultrasonic microreactor, we compared the physicochemical and optoelectronic properties of the obtained HPQDs with those prepared by representative conventional routes. Conventional batch ligand-assisted reprecipitation (LARP) and template-regulation methods are fundamentally limited by relatively slow and non-uniform convective mass transfer, which inevitably generates local supersaturation gradients and asynchronous nucleation events. These macroscopic fluidic limitations generally lead to broader size distributions and severe inhomogeneous photoluminescence broadening. For instance, conventional ambient batch syntheses without sophisticated kinetic control typically suffer from severe size defocusing, yielding broad full width at half maximum (FWHM) values up to 41–53 nm [10], [45], and even advanced thermodynamic or chemical coordination routes in batch phases often require tedious post-synthetic processing to compress the emission linewidth to approximately 25–31 nm [10], [45]. In sharp contrast, the present ultrasonic microreactor seamlessly combines continuous-flow microfluidic confinement with localized acoustic cavitation, intense acoustic streaming, and intensified micro-convection. These collaborative sonochemical effects drastically shorten the characteristic mixing time well below the rapid nucleation threshold and promote a highly homogeneous supersaturation field, leading to synchronized burst nucleation and restricted secondary growth. Accordingly, the HPQDs obtained in this work directly exhibit exceptionally narrow, in-situ emission linewidths of 23.28–30 nm without requiring any tedious post-synthetic size selection.

In addition to size and spectral uniformity, the ultrasonic microreactor significantly improves crystallinity and surface passivation. In conventional ambient syntheses governed by dynamic ligand pairs, disordered ligand adsorption/desorption typically leaves a high density of under-coordinated metal sites and halide vacancies, resulting in dense sub-bandgap surface trap states and fatal non-radiative recombination. The enhanced mass transfer and high-velocity micro-jets within the ultrasonic microreactor facilitate a highly efficient, in-situ ligand chemisorption during the earliest stages of nanocrystal formation, thereby effectively stabilizing the crystal lattice and reducing defect density from the root. This is robustly supported by the enhanced photoluminescence quantum yield (PLQY), and prolonged time-resolved photoluminescence (TRPL) lifetime. Crucially, without requiring any sophisticated physical encapsulation or high-temperature glass melting, the optimized HPQDs exhibit an outstanding peak PLQY of 78% under mild ambient conditions, directly outperforming representative advanced batch-derived benchmarks such as modified LARP systems (46%) [46], rare-earth modified borosilicate glass networks (53.79%) [47], and dual-functional porous frameworks (75.8%) [48]. Furthermore, the reduced fast-decay component in the TRPL spectra indicates successfully suppressed trap-assisted non-radiative recombination. These results collectively demonstrate that the ultrasonic microreactor does not merely accelerate synthesis, but also provides improved control over nucleation, growth, surface passivation, and carrier recombination dynamics.

To firmly elucidate the fluidic origins underpinning this unprecedented control over crystallographic and optoelectronic properties, it is essential to validate the reliability of these simulated flow fields and establish their direct correlation with experiential outcomes, the optimized structural parameters derived from the flow behavior were translated into physical reactor designs. The simulations indicate that the cylindrical microstructure provides a more balanced flow environment, enhancing local mixing and boundary-layer disruption while avoiding excessive flow instability. Guided by this result, the optimized cylindrical reactor with *H* = 0.2 mm and *d* = 0.8 mm was used experimentally. This configuration produced an approximately 11-fold enhancement in 3D PL intensity and a high absolute PLQY of 78.6%, which is consistent with improved mixing and mass transfer predicted by the simulations.

#### 3.2.2 Effect of precursor flow rate on HPQDs spectroscopic performance in the optimized sharp-edged ultrasonic microreactor

Precursor flow rate is a core operating parameter that governs reaction residence time and precursor mixing stoichiometry in continuous-flow microreactors, enabling deterministic tuning of HPQDs morphology and optical properties. All experiments were performed at room temperature with fixed operating parameters: ultrasonic frequency of 21 kHz, ultrasonic power of 100 W, and precursor solution B flow rate of 1 mL/min. The flow rate of precursor solution A was systematically varied across a gradient of 100–300 μL/min (100, 150, 200, 250, and 300 μL/min) to characterize its regulatory effect on HPQDs spectroscopic performance. Fig. 8 reveals the deterministic regulatory effect of precursor flow rate on HPQDs optoelectronic properties, with the PL emission peak exhibiting a distinct red-shift followed by blue-shift trend (Fig. 8c) — a direct manifestation of flow rate-dependent crystal nucleation and growth kinetics.

At the lowest flow rate (100 μL/min), the PL emission peak centers at 515 nm (Fig. 8d) with a relatively broad FWHM (>28 nm). This inferior spectral performance arises from the mixing-limited regime at low Reynolds numbers (*Re* ≈ 800–1200): laminar-dominated flow restricts the perturbation range of ultrasonic cavitation microjets, hindering local precursor mass transfer and leading to heterogeneous crystal nucleation and growth. The resulting polydisperse HPQDs and abundant surface trap states further broaden the FWHM and weaken luminescence intensity. Increasing the flow rate to 150 μL/min drives the flow into a transitional turbulence regime, where intensified turbulent shear forces disrupt the laminar diffusion boundary layer. This promotes rapid, uniform precursor mixing and high reaction conversion, facilitating effective Ostwald ripening — where smaller, less stable nanocrystals dissolve and larger, thermodynamically stable ones grow. Consequently, the PL emission peak red-shifts to 521 nm, and the FWHM narrows to a minimum of 23.28 nm, marking the optimal thermodynamic equilibrium between mixing efficiency and crystal growth residence time. This configuration yields HPQDs with the highest monodispersity and crystallinity, corroborated by complementary spectroscopic characterization. Further increasing the flow rate to 300 μL/min pushes the reaction into a residence-time-limited regime. Crystal nuclei are prematurely transported out of the reaction zone immediately after formation, forcibly truncating the growth process via a so-called kinetic freezing effect. While this effect reduces the average HPQDs size, it also drives an abrupt blue-shift of the PL emission peak to 502 nm. More critically, kinetic freezing results in incomplete crystal growth and increased surface trap density, as evidenced by the significant attenuation of PLE intensity (Fig. 8b) and degraded crystalline quality. This dynamic, parameter-driven trajectory of spectral evolution is fundamentally underpinned by the rigorous hydrodynamic stability of the microfluidic coupling field: as statistically quantified (n ≥ 3) in Fig. 8d, although the migrating FWHM and shifting emission peaks evolve regularly with convective mass transfer, the replication variance at each discrete operational point remains rigorously compressed, keeping the average FWHM RSD below 8.5% within the core 100–200 μL/min regime. This exceptionally narrow error envelope proves that the system successfully buffers external mechanical shear fluctuations, establishing a time-invariant physical microenvironment that ensures remarkable batch-to-batch reproducibility.

UV-vis absorption spectra further validate the optimal flow rate (Fig. 8a): the 150 μL/min sample exhibits the steepest, most well-defined excitonic absorption peak, indicative of high crystallinity and low defect density. In contrast, the low-flow group (100 μL/min) shows a high-scattering baseline, attributed to heterogeneous HPQDs aggregation, while the high-flow group (300 μL/min) presents a significant blue-shift of the absorption edge, consistent with reduced HPQDs size and quantum confinement. Optical photographs (Fig. 8e) visually corroborate a gradient in emission color, from bright emerald green (150 μL/min) to deep blue (300 μL/min), confirming flow rate modulation as a robust tool to tune the quantum confinement effect and luminescence brightness of HPQDs.

**Fig. 9:**
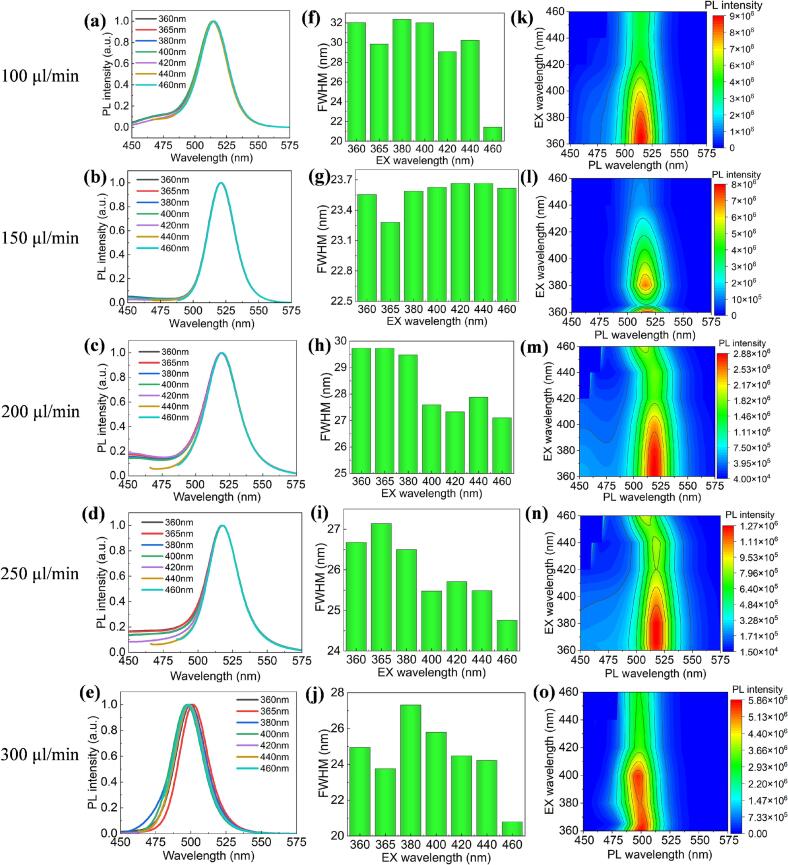
Flow rate-dependent excitation wavelength stability of HPQDs synthesized in the optimized sharp-edged ultrasonic microreactor. All syntheses are performed under strictly fixed operating conditions: ultrasonic excitation (21 kHz, 100 W), precursor solution B flow rate of 1 mL/min, with precursor solution A flow rate systematically varied across 100, 150, 200, 250, and 300 μL/min. (a–e) 2D normalized PL spectra of HPQDs at each flow rate, recorded across excitation wavelengths of 360–460 nm; (f–j) Quantitative statistics of PL FWHM as a function of excitation wavelength (360–460 nm) for the corresponding samples; (k–o) 3D PL spectra of HPQDs at each flow rate, acquired across 360–480 nm excitation wavelengths.

Fig. 9 presents detailed excitation wavelength-dependent PL characterization of HPQDs across 360–460 nm excitation. At a lower flow rate of 100 μL/min, the HPQDs exhibit a noticeable fluctuations in their optical symmetry and color purity. As illustrated in the 2D normalized PL spectra (Fig. 9a) and the corresponding 3D PL contour plot (Fig. 9k), the sample delivers a compromised PL intensity. The quantitative statistics (Fig. 9f) further reveal a volatile full-width at half-maximum (FWHM) ranging from 21 to 32 nm across different excitation wavelengths (360–460 nm). This optical heterogeneity is rationally ascribed to the prolonged residence time under low flow velocity, which induces severe back-mixing and thermal fluctuations from acoustic cavitation, thereby precipitating over-growth and broad size distribution of the nanocrystals.

In sharp contrast, establishing the flow rate at 150 μL/min emerges as the optimal regimes, unlocking exceptional spectral resolution and maximum quantum yield through precise sonochemical regulation. HPQDs exhibit near-ideal excitation wavelength independence: regardless of excitation energy, their 2D emission spectra (Fig. 9b) show a consistent peak centered at 521 nm, with FWHM (Fig. 9g) stably confined to an ultra-narrow range. The corresponding 3D emission spectrum (Fig. 9l) presents a high-intensity, highly symmetric bullseye topological structure, which definitively demonstrates that the uniform turbulent field generated by the optimized sharp-edged microstructures enables homogeneous confined nucleation of reactant molecules at this flow rate. This homogeneous nucleation effectively suppresses deep-level surface traps and mitigates size polydispersity, the critical prerequisites for stable, high-performance optical output.

When the flow rate transcends this threshold into the higher regimes of 200, 250, and 300 μL/min, the optical quality undergoes a progressive degradation driven by mass-transfer and kinetic limitations. Within the intermediate-high range of 200 and 250 μL/min, although the 2D spectra (Fig. 9c, d) still maintain an excitation-independent emission at 518 nm, their respective FWHM trends (Fig. 9h, i) begin to distort and fluctuate (24–30 nm), accompanied by a steady decline in the 3D PL intensities (Fig. 9m, n). Upon accelerating further to 300 μL/min, a catastrophic collapse in optical properties is observed: the 2D emission band (Fig. 9e) experiences a significant blue-shift to 502 nm, the FWHM profile (Fig. 9j) behaves erratically, and the 3D PL mapping (Fig. 9o) undergoes a severe intensity attenuation down. This holistic deterioration at high flow velocities is fundamentally attributed to the drastic reduction of effective residence time within the active acoustic zone. The rapid fluid transit prematurely truncates the growth stage of the nuclei and dampens the acoustic energy density absorbed per unit volume, which ultimately impedes the sonochemical crystallization and amplifies defect-induced non-radiative recombination pathways.

#### 3.2.3 Effect of precursor concentration on HPQDs spectroscopic performance in the optimized sharp-edged ultrasonic microreactor

Precursor concentration is a core parameter governing HPQDs nucleation and growth kinetics, enabling deterministic tuning of nanocrystal morphology and optoelectronic properties. To characterize its regulatory effect, we set precursor concentration as the sole experimental variable, with all other operating parameters fixed: room temperature, precursor solution A flow rate of 150 μL/min, and continuous ultrasonic excitation (21 kHz, 100 W). Precursor concentration was systematically varied across 0.2–1.0 mol/L to investigate its impact on the morphological and spectroscopic properties of HPQDs synthesized in our optimized sharp-edged ultrasonic microreactor.

**Fig. 10:**
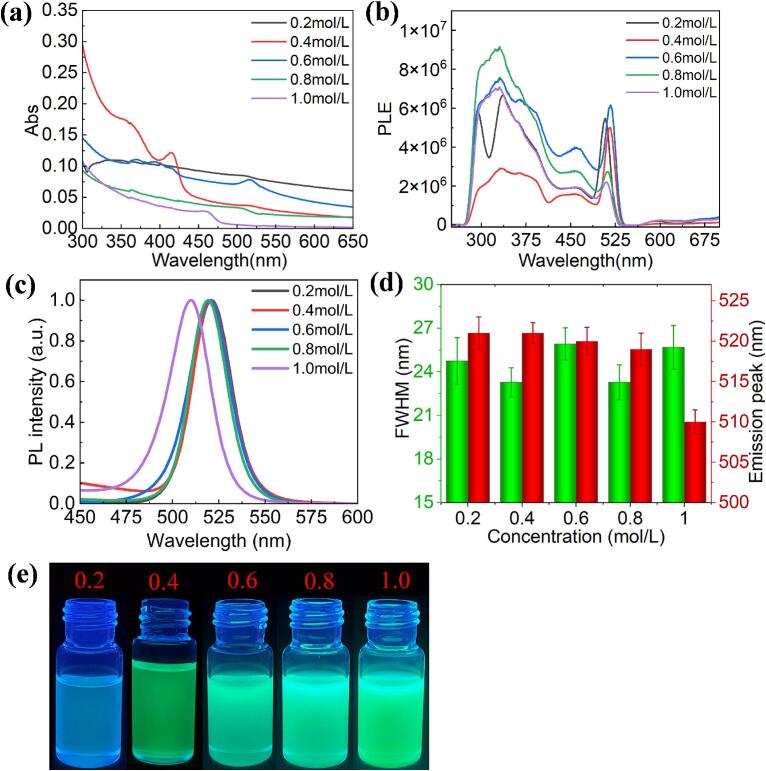
Optoelectronic characterization of HPQDs synthesized at varying precursor concentrations in the optimized sharp-edged ultrasonic microreactor. (a) UV–vis absorption spectra; (b) PLE spectra; (c) Steady-state PL emission spectra; (d) Corresponding PL FWHM and emission peak position statistics; (e) Fluorescence photographs of HPQDs solutions under 365 nm UV irradiation.

Fig. 10 reveals the deterministic regulatory effect of precursor concentration on HPQDs crystal monodispersity and optoelectronic performance. PL emission spectra (Fig. 10c) show that in the low-to-moderate concentration range (0.2–0.6 mol/L), the emission peak remains stable at ∼521 nm. The corresponding UV-Vis absorption spectra (Fig. 10a) exhibit steep, well-defined excitonic absorption peaks, indicative of high crystallinity and low defect density. Complementing these observations, the PLE spectra (Fig. 10b) manifest the most robust excitation efficiency and peak symmetry at 0.4 mol/L, with a minimized Stokes shift that underscores an optimized electronic density of states. This superior excitation profile, combined with the FWHM trend (Fig. 10d) reaching a minimum of 23.28 nm, demonstrates an optimal match between the ultrasonic cavitation shear forces, amplified by our optimized sharp-edged microstructures, and precursor supersaturation at 0.4 mol/L: moderate supersaturation triggers homogeneous confined nucleation, while sufficient monomer supply sustains subsequent size-focusing growth, yielding HPQDs with exceptional size monodispersity. This concentration-governed crystallization determinism is fundamentally supported by the rigorous chemical kinetics tolerance and boundary metrics compiled in Fig. 10d. Across the 0.2–1.0 mol/L concentration gradient, both the migrating FWHM (green bars) and the shifting emission peaks (red bars) follow highly reproducible thermodynamic pathways rather than stochastic drifting. At the optimized concentration of 0.4 mol/L, the system achieves its tightest batch control, confining the replication standard deviation to a minimal SD of ± 1.0 nm (RSD = 4.3%), providing concrete empirical proof of deterministic regulation. Conversely, at the extreme concentration limit of 1.0 mol/L, the replication variance expectedly widens (SD = ± 3.9 nm), which successfully maps the physical boundary of our control envelope where severe acoustic attenuation dominates over sonochemical regulation due to heightened fluid viscosity.

In contrast, increasing the precursor concentration to 1.0 mol/L generates excessive supersaturation, which triggers uncontrolled burst nucleation and increases the solution's viscosity, thereby inducing a significant “viscous dampening” effect on acoustic energy dissipation. This dampening weakens the sonochemical passivation of surface states, as evidenced by the diminished fine structures in the PLE spectra (Fig. 10b). Such a regime instantaneously depletes free monomers and arrests crystal growth at an early stage, driving a blue shift of the PL emission peak to 510 nm. Consistent with these spectral changes, optical photographs (Fig. 10e) show that the HPQDs solution transitions from a clear, bright emerald green at 0.4 mol/L to a turbid, dull cyan at 1.0 mol/L, confirming that extreme supersaturation induces severe HPQDs agglomeration and associated Mie scattering.

**Fig. 11:**
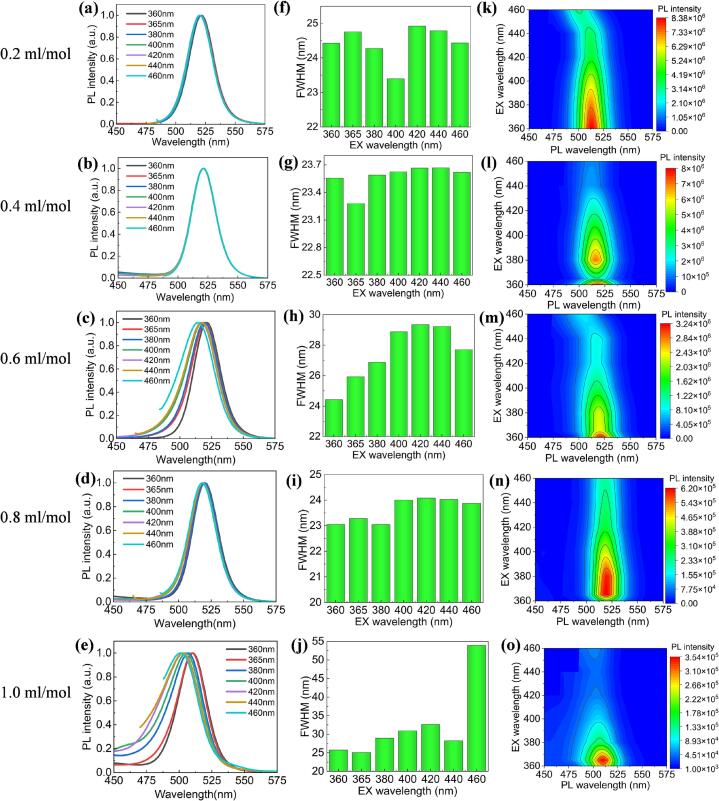
Excitation wavelength-dependent PL characterization of HPQDs synthesized at varying precursor concentrations. All samples are prepared in the optimized sharp-edged ultrasonic microreactor under fixed operating conditions (ultrasonic excitation: 21 kHz, 100 W; precursor solution A flow rate: 150 μL/min), with precursor concentration varied across 0.2, 0.4, 0.6, 0.8, and 1.0 mol/L. (a–e) 2D normalized PL spectra of HPQDs at the above concentrations, acquired across excitation wavelengths of 360–460 nm; (f–j) Corresponding PL FWHM as a function of excitation wavelength (360–460 nm) for each concentration; (k–o) 3D PL spectra of HPQDs at the above concentrations, acquired across 360–480 nm excitation wavelengths.

Fig. 11 presents a detailed excitation wavelength-dependent PL characterization of the synthesized HPQDs across an excitation range of 360–460 nm under varying precursor concentrations from 0.2 to 1.0 mol/L. At a low precursor concentration of 0.2 mol/L, the HPQDs display minor variations in their overall emission features. As shown in the 2D normalized PL spectra (Fig. 11a) and the corresponding 3D PL contour plot (Fig. 11k), the sample provides a relatively low PL intensity. The quantitative statistics (Fig. 11f) reveal that the FWHM values fluctuate between 23.3 and 25.0 nm across the tested excitation wavelengths. This behavioral fluctuation is rationally ascribed to the insufficient chemical potential and lower collision frequency of the reaction monomers under a dilute state. In this regime, the chemical driving force for nucleation is deficient, which mildly disrupts the synchronization of crystal growth despite the intense mass transfer provided by acoustic cavitation.

In sharp contrast, establishing the precursor concentration at 0.4 mol/L emerges as the optimal regime, unlocking exceptional spectral resolution and maximum emission efficiency through precise sonochemical regulation. The 2D PL spectra (Fig. 11b) display an impeccable overlap with strict excitation-wavelength independence, pinning the emission center precisely at 521 nm. This structural and optical homogeneity is rigorously validated by the FWHM plot (Fig. 11g), where the values are remarkably stabilized at a global minimum of 23.56 nm, irrespective of the excitation wavelength. Crucially, the 3D PL mapping (Fig. 11l) presents a highly symmetric, narrow emission profile with a towering peak intensity, outperforming all other concentration counterparts by two to three orders of magnitude. This profound enhancement indicates that at 0.4 mol/L, the chemical reaction thermodynamics perfectly synchronize with the physical acoustic field. Under this synergistic state, the precursor concentration exactly satisfies the threshold for “burst nucleation” triggered by ultrasonic waves, allowing the robust acoustic streaming to uniformly distribute the chemical supersaturation, thereby facilitating ideal synchronized nucleation while executing thorough surface passivation to eliminate deep-level non-radiative trap states.

When the precursor concentration transcends this threshold into the higher regimes of 0.6, 0.8, and 1.0 mol/L, the optical quality undergoes a progressive and severe degradation driven by viscous dampening of acoustic energy and uncontrolled over-saturation kinetics. Within the intermediate-high range of 0.6 and 0.8 mol/L, the 2D spectra (Fig. 11c, d) begin to exhibit a distinct excitation-dependent emission shift, accompanied by a noticeable broadening in their respective FWHM trends to 24–29.5 nm (Fig. 11h) and 23–24 nm (Fig. 11i). Simultaneously, the 3D PL intensities steadily decline (Fig. 11m, n). Upon accelerating further to 1.0 mol/L, a catastrophic collapse in optical properties is observed: the 2D emission band (Fig. 11e) experiences a substantial excitation-dependent shift and curve splitting, the FWHM profile (Fig. 11j) behaves erratically with a dramatic surge to 54 nm at 460 nm excitation, and the 3D PL mapping (Fig. 11o) undergoes a severe intensity attenuation down.This holistic deterioration at high concentrations is fundamentally distinct from flow-rate configurations; it is attributed to the increased viscosity of the concentrated precursor solution, which severely dampens the acoustic cavitation intensity and restricts energy dissipation. The weakened acoustic fields can no longer suppress the intrinsic, chaotic mass-action kinetics of high-concentration solutions. Consequently, excessive local supersaturation triggers heterogeneous, non-synchronized spontaneous nucleation and severe particle agglomeration, which fundamentally compromises the sonochemical crystallization process and amplifies defect-induced non-radiative recombination pathways.

Beyond validating these microscopic structural and photophysical optimization mechanisms, translating bench-scale nanocrystal regulation into highly stable and high-throughput manufacturing is a prerequisite for real-world technological deployment. The optimized ultrasonic microreactor provides continuous HPQDs production with quantified single-channel productivity and stable 24 h operation. During continuous synthesis, the PLQY remained at approximately 78.1% with an RSD of 2.4%, while the emission peak drift was confined within ±0.5 nm. The product recovery remained approximately 94.2%, and no obvious channel blockage was observed during the tested period. Furthermore, a 10-channel parallelized microreactor was demonstrated as a numbering-up strategy, achieving a scale-up factor of 10 while maintaining inter-channel FWHM variation below 3.5%. These results support the practical scalability and operational reliability of the ultrasonic microreactor within the tested operating window.

## 4 Conclusions

In this work, we have developed a biomimetic vein-inspired ultrasonic microreactor integrated with sharp-edged microstructure arrays for high-efficiency, continuous, and deterministic synthesis of HPQDs. Through a tightly coupled framework of multiphysics numerical simulation and experimental validation, we systematically establish the quantitative structure-performance relationship between microstructure geometric parameters, hydrodynamic behavior, and sonochemical effects in the microreactor. Using fluid dynamics and acoustic field simulations, we comprehensively decode the impacts of sharp-edged morphology, height, and inter-structure spacing on flow field characteristics and cavitation distribution, identifying a cylindrical microstructure (height: 0.2 mm, spacing: 0.8 mm) as the globally optimal geometric configuration. This design synergistically amplifies acoustic streaming intensity and cavitation yield within the microchannel, drastically enhancing multiphase micromixing efficiency and creating a well-defined thermodynamic and kinetic environment for high-quality nanocrystal nucleation and growth — directly overcoming the longstanding mass transfer limitation bottleneck inherent to conventional laminar microreactors.

Leveraging this optimized microreactor configuration, we successfully synthesize high-performance HPQDs with an narrow FWHM as low as 23.28 nm and a PLQY up to 78.6%, markedly outperforming state-of-the-art conventional microfluidic synthesis methods. Multidimensional characterization reveals the fundamental mechanism of ultrasonic performance enhancement: cavitation-generated shear forces and transient high-temperature/high-pressure environments promote homogeneous precursor nucleation and lattice perfection, while effectively passivating surface trap states. Furthermore, precise tuning of precursor flow rate and concentration achieves dynamic supersaturation equilibrium in the reaction system, enabling controlled size focusing as defined by the LaMer model and ensuring exceptional HPQDs size monodispersity.

In summary, this work presents an efficient biomimetic ultrasonic microfluidic synthesis strategy, which offers significant advantages in mass transfer intensification, agglomeration inhibition, and continuous scalable production. It also provides a new mechanistic perspective for understanding nanocrystal nucleation-growth dynamics under acoustic-hydrodynamic coupling, with broad application prospects for low-cost, scalable manufacturing of high-performance optoelectronic nanomaterials.

## CRediT authorship contribution statement

**Shengxin Zhu:** Writing – original draft, Visualization, Methodology, Investigation, Data curation. **Jianwei Liao:** Visualization, Data curation. **Longshi Rao:** Writing – review & editing, Supervision, Methodology, Funding acquisition, Conceptualization. **Yuying Wang:** Visualization, Data curation. **Xiang Huo:** Visualization, Investigation, Data curation. **Qinghao Zhong:** Visualization, Data curation. **Haoyu Chen:** Visualization, Investigation, Data curation. **Guisheng Zhong:** Writing – review & editing, Investigation, Funding acquisition. **Xiaodong Niu:** Validation, Supervision, Methodology.

## Declaration of competing interest

The authors declare that they have no known competing financial interests or personal relationships that could have appeared to influence the work reported in this paper.

## Appendix A Supplementary data

Supplementary Data 1 Calculation of Reynolds number, mass transfer scaling & mixing efficiency formulations, theoretical decoupling and synergistic mechanism of acoustic streaming and inertial cavitation, supplementary figures, and additional tables.
