# Supplementary material for: Simulation-guided biomimetic sharp-edged ultrasonic microreactor enables morphology-uniform and performance-tunable halide perovskite quantum dots
Source: Ultrason Sonochem. 2026 Jul 3;132:107948. doi: 10.1016/j.ultsonch.2026.107948 (PMC13380530; doi:10.1016/j.ultsonch.2026.107948)
Supplement: Supplementary Data 1 — Calculation of Reynolds number, mass transfer scaling & mixing efficiency formulations, theoretical decoupling and synergistic mechanism of acoustic streaming and inertial cavitation, supplementary figures, and additional tables. [file mmc1.docx]

**Simulation-guided biomimetic sharp-edged ultrasonic microreactor enables morphology-uniform and performance-tunable halide perovskite quantum dots**

**Shengxin Zhu^1,2^, Jianwei Liao^1,2^,** **Longshi Rao^1,2,3*^, Yuying Wang^1,2^, Xiang Huo^1,2^, Qinghao Zhong^1,2^, Haoyu Chen^1,2^, Guisheng Zhong^1,2,4^, and Xiaodong Niu^1,2^**

^1^ Department of Mechanical Engineering, College of Engineering, Shantou University, Shantou 515063, China

^2^ Intelligent Manufacturing Key Laboratory of Ministry of Education, Shantou University, Shantou 515063, China

^3^ Guangdong Provincial Key Laboratory of Automotive Display and Touch Technologies, Shantou Goworld Display Technology Co., Ltd., Shantou 515041, China

^4^ Shantou Key Laboratory for Intelligent Equipment and Technology, Shantou University, Shantou 515063, China

*Corresponding author: lsrao@stu.edu.cn.

**SI 1 Reynolds number (*R*_e_)**

**(1)** Mean velocity (*U*) is determined by:

$$U=Q/\left(D\bullet h\right)$$

Where *Q* is the volumetric flow rate, *D* and *h* is the width depth of the channel inlet, respectively.

**(2)** Reynolds number (*R*_e_) is calculated using the formula:

$$R_{e}=\rho\bullet U\bullet D_{h}/\mu$$

where *ρ* is the density of C_6_H_5_Cl, *D_h_* is the hydraulic diameter, and *μ* is the viscosity of C_6_H_5_Cl.

Since the properties of C_6_H_5_Cl dominate the mixture, the non-dimensional numbers were calculated using C_6_H_5_Cl as follows:

**(3) Assumptions / input**

- Solvent approximated by C_6_H_5_Cl -rich properties (as requested): *ρ* = 1.107×10³ kg·m⁻³; *μ* = 0.799×10⁻³ Pa·s (25 °C).
- Channel inlet: width *D* = 1 mm (0.001 m); depth *h* = 0.4 mm.
- Volumetric flow rates *Q* =1 mL·min⁻¹.
- Hydraulic diameter (rectangular): *D_h_*_​_ = 2*D·h*/(*D* + *h*).
- Formulas used: *U* = *Q*/(*D·h*); *R_e_* = *ρ·U·D_h_*​/*μ*; Streaming Reynolds *R_es_*​ ≈ *U_s_*​*·D_h_*_​_/*ν* (requires an acoustic streaming velocity amplitude *Us*​).

**(4) Representative computed values**

- Hydraulic diameter *D_h_*​:

*D_h_*​ = 5.71×10⁻^4^ m;

- Mean velocity *U* (m·s⁻¹):

*U*=0.04167 m·s⁻¹ (*Q* = 1 mL·min⁻¹);

- Reynolds number:

*R*_e_= *ρ·U·D_h_*​/*μ*: ≈ 32.99

- Streaming / acoustic Reynolds (order estimate): *R_es_*​ ≈ *U_s_*​*·D_h_*​/*ν*. Using plausible streaming velocities *U_s_*_​_ = 1.2 m·s⁻¹ (typical lab streaming amplitudes reported in the literature) and *ν* = *μ*/*ρ* ≈ 7.22×10⁻⁷ m²·s⁻¹ yields *R_es_*​ ≈ 949.

*Conclusion:* realistic acoustic streaming amplitudes produce local *R_es_*_​_ large enough to generate vortices and strong micro-mixing even though bulk *R*_e_ is small.

**SI 2 Mass Transfer Scaling & Mixing Efficiency Formulations**

To quantify the convective mass transfer intensification under 21 kHz ultrasound, the dimensionless Péclet number (*P_e_*) and the cross-sectional Coefficient of Variation (*CoV*) are evaluated.

1. **Péclet Number (*P_e_*):**

$$Pe=\frac{\text{Convective Transport}}{\text{Diffusive Transport}}=\frac{U_{s}\cdot D_{h}}{D_{m}}$$

Where *D_m_*≈1.2×10^-9^ m^2^·s⁻¹ represents the molecular diffusion coefficient of the reactive precursor solutes.

- Without Ultrasound: *P_e_* is strictly diffusion-limited across parallel streams.
- With Ultrasound: Driven by the intense acoustic streaming velocity (*U_s_* = 1.2 m·s⁻¹), the local *P_e_* scales as:

$$Pe_{acoustic}=\frac{1.2\text{ m}\cdot\text{s}^{-1}\times5.71\times10^{-4}\text{ m}}{1.2\times10^{-9}\text{ m}^{2}\cdot\text{s}^{-1}}\approx5.71\times10^{5}$$

This drops the mass transfer scaling resistance by over three orders of magnitude compared to the pure diffusion state, switching the transport mechanism to immediate chaotic advection.

1. **Coefficient of Variation (*CoV*):**

The micromanifold mixing homogeneity across the channel cross-section is quantified via the concentration distribution field:

$$CoV=\left(1-\frac{\sigma_{c}}{\bar{c}}\right)\times100\%=\left(1-\frac{\sqrt{\frac{1}{N}\sum_{i=1}^{N} (c_{i}-\bar{c})^{2}}}{\bar{c}}\right)\times100\%$$

Where *c_i_* is the localized nodal concentration, *$\bar{c}$* is the mean cross-sectional concentration, and $\sigma_{c}$ is the standard deviation. Driven by 21 kHz acoustic fields, the local *CoV* dynamically surges from a baseline of 34.2% to 98.1% within milliseconds.

**SI 3 Theoretical decoupling and synergistic mechanism of acoustic streaming and inertial cavitation**

Due to micro-scale geometric constraints, direct in-situ diagnostics (e.g., sonoluminescence) are restricted. To rigorously distinguish the physical boundaries and roles of acoustic streaming (AS) and inertial cavitation (IC) in enabling deterministic HPQDs synthesis, a condensed multi-scale scaling and governing equation analysis under the 21 kHz field is established.

1. **Multi-Scale Scaling Disambiguation**

AS and IC are driven by the same 21 kHz field but operate on fundamentally decoupled spatial, temporal, and frequency scales, as quantified in Table S1.

Table S1. Quantitative physical boundaries and criteria for decoupling AS and IC.

| Physical Feature | Acoustic Streaming (AS) | Inertial Cavitation (IC) | Decoupling Criterion |
| --- | --- | --- | --- |
| Driving Mechanism | Time-averaged Reynolds stress gradient (*F_s_​*) | Peak negative transient pressure (*P_neg_*​>*P_th​_*) | Hydrodynamic force vs. Fluid phase rupture |
| Spatial Scale | Hydraulic diameter (*D_h_*​≈5.71×10^−4^ m) | Bubble collapse radius (*R_b_​*∼1–10 μm) | Macro-convective vs. Micro-interfacial |
| Temporal Scale | Continuous steady-state (*τ_AS_​*∼ms) | Transient impulse duration (τ_IC​_∼μs) | Steady advection vs. Transient shock |
| Acoustic Frequency | 21 kHz (Long advection response time) | 21 kHz (Long acoustic period allows large bubble expansion) | Low frequency intensifies inertial cavitation violence |
| Functional Role | Bulk fluid homogenization (*CoV*→98.1%) | Lowering crystallization energy barriers via hotspots | Macroscopic mixing vs. Burst nucleation |

1. **Governing Equations and Scale Separation**
2. **Acoustic Streaming (Macro-Convective Mixing)**

Acoustic streaming is governed by the time-averaged incompressible Navier-Stokes equations, where the MHz acoustic field enters exclusively as a secondary quality body force (*F_s_*):

$$\rho_{0}(\mathbf{u}_{s}\cdot\nabla)\mathbf{u}_{s}=-\nabla p_{s}+\mu\nabla^{2}\mathbf{u}_{s}+\mathbf{F}_{s}, \nabla\cdot\mathbf{u}_{s}=0$$

$$\mathbf{F}_{s}=-\rho_{0}\nabla\cdot\langle\mathbf{v}_{1}\mathbf{v}_{1}\rangle$$

Where *u_s_* is the steady streaming velocity and *v_1_* is the first-order acoustic velocity. The evaluated streaming Reynolds number (R_es_≈949) confirms that AS provides massive macro-convective inertia to break down parallel laminar streamlines for bulk fluid homogenization.

1. **Inertial Cavitation (Micro-Interfacial Hotspots)**

Inertial cavitation is a highly localized, high-energy-density phase-rupture event. At 21 kHz, the long acoustic period (~47.6 μs) allows micro-bubbles sufficient time to accumulate mass and expand. Driven by localized pressure peaks exceeding the solvent threshold (*P_neg_*≈ 2.4 MPa > *P_th_*≈1.0-1.5 MPa), the dynamic radius (*R*) is governed by the Rayleigh-Plesset equation:

$$R\frac{d^{2}R}{dt^{2}}+\frac{3}{2}\left(\frac{dR}{dt}\right)^{2}=\frac{1}{\rho_{0}}\left[\left(P_{0}+\frac{2\sigma}{R_{0}}-P_{v}\right)\left(\frac{R_{0}}{R}\right)^{3\gamma}+P_{v}-\frac{2\sigma}{R}-\frac{4\mu}{R}\frac{dR}{dt}-P_{\infty}(t)\right]$$

The violent asymmetric bubble collapse near solid boundaries fires high-speed liquid micro-jets (Ujet > 100 m·s⁻¹) and generates transient mechanical energy ($\Delta$G_acoustic_), which directly lowers the critical crystallization nucleation energy barrier ($\Delta$G^*^):

$$\Delta G^{*}=\frac{16\pi\sigma^{3}}{3(\Delta G_{v}-\Delta G_{acoustic})^{2}}$$

1. **Unified Synergistic Mechanism**

The multi-scale synergy operates sequentially to enable deterministic synthesis control:

**1. Stage 1 (AS, ~scale):** Continuous high-velocity streaming jets (*U_s_* = 1.2 m·s⁻¹) induce rapid cross-stream advection, achieving ultra-uniform reactant homogenization (*CoV* = 98.1%) and eliminating macro-concentration gradients.

**2. Stage 2 (IC, ~ μs scale):** Within the homogenized matrix, the highly violent bubble collapses inherent to 21 kHz low-frequency ultrasound input localized mechanical shocks at the precursor reaction interfaces. This thermodynamic shock triggers an instantaneous, synchronized burst nucleation of HPQDs, ensuring narrow photoluminescence emission linewidths.


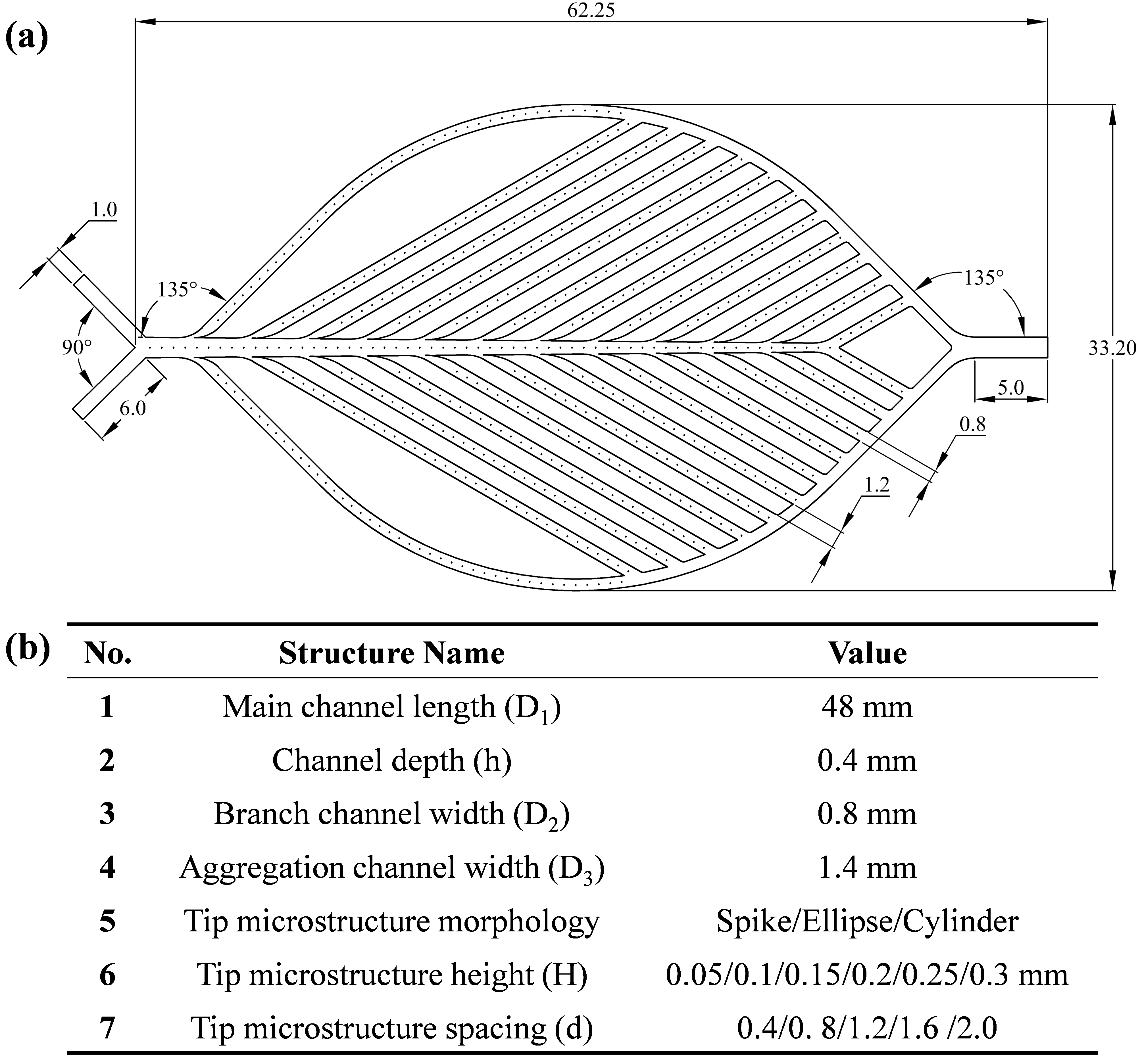


**Fig. S1.** (a) Dimensional diagram of the vein-inspired microchannel integrated with sharp-edged microstructures. (b) Schematic of the key structural parameters for the sharp-edged, vein-inspired microchannel.


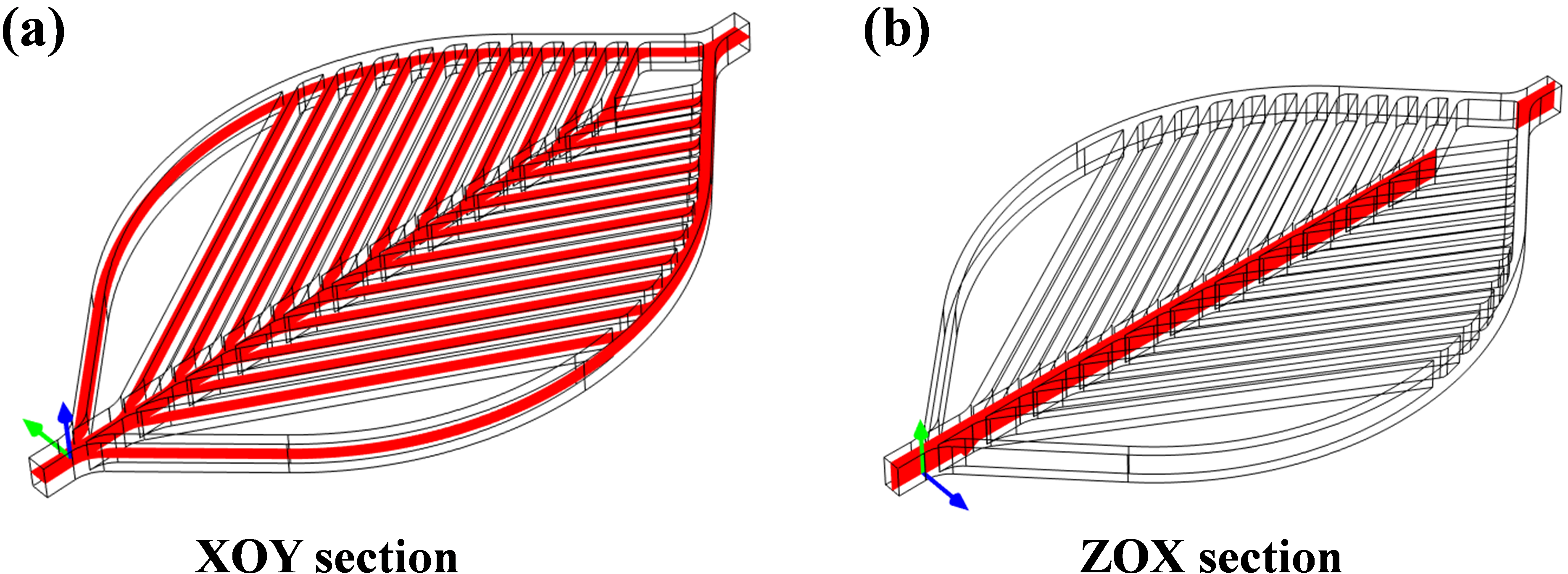


**Fig. S2.** (a) XOY plane: Top view of the microchannel. (b) ZOX plane: Front view of the main channel.

**Table S2.** Pressure distribution contours on the XOY plane for microchannels with different sharp-edged morphologies.


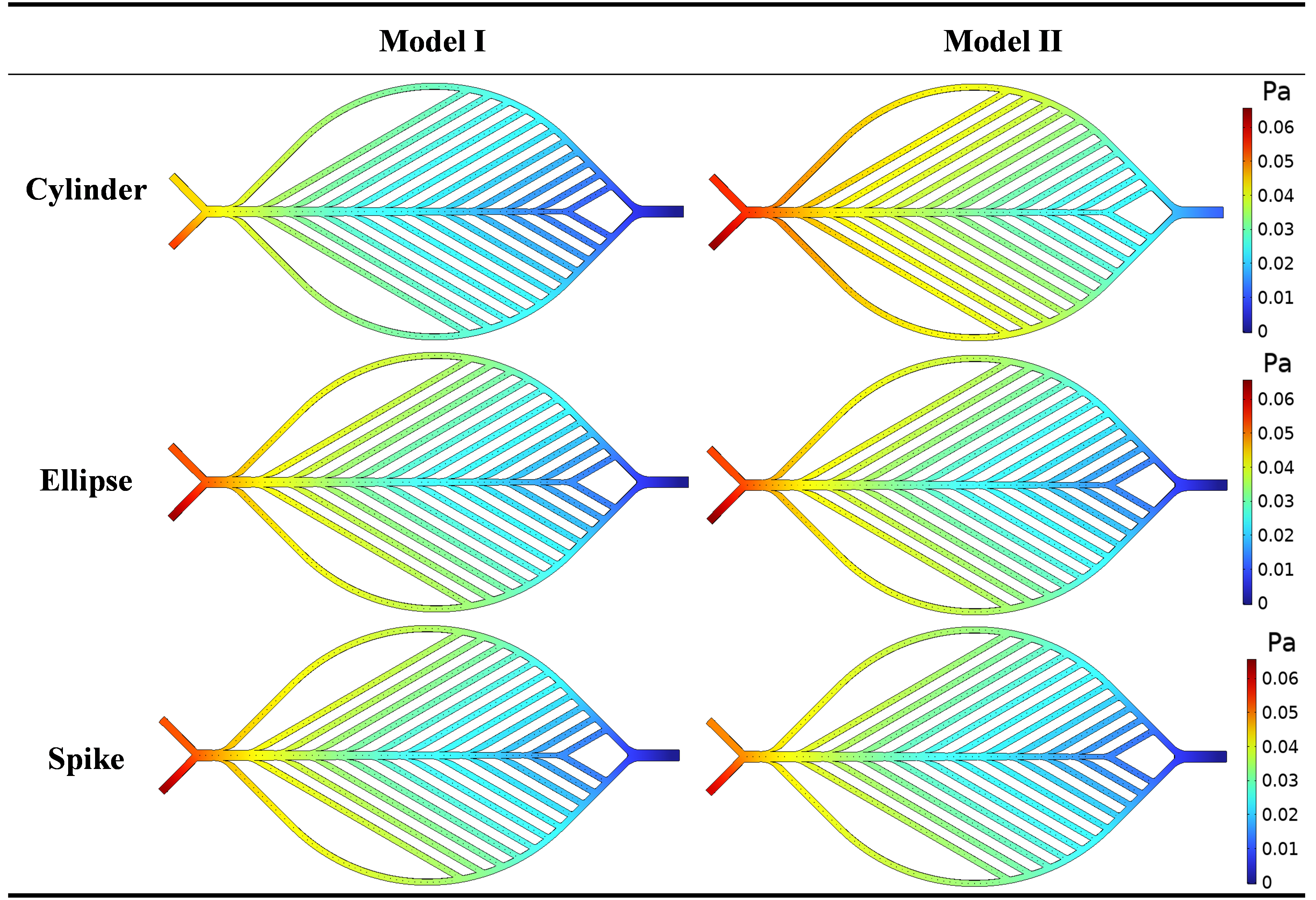


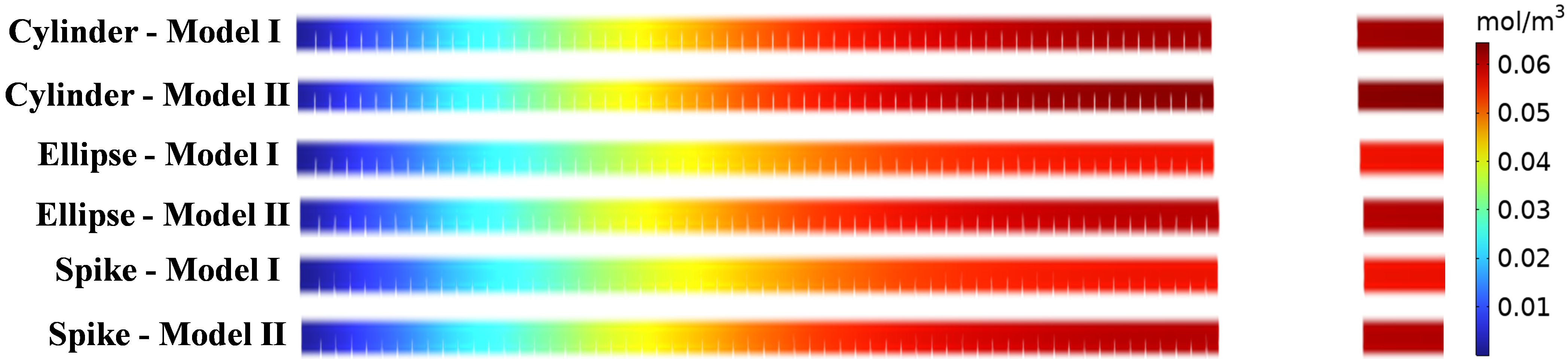


**Fig. S3.** HPQDs concentration distribution maps on the ZOX plane for microchannels with different sharp-edged morphologies.


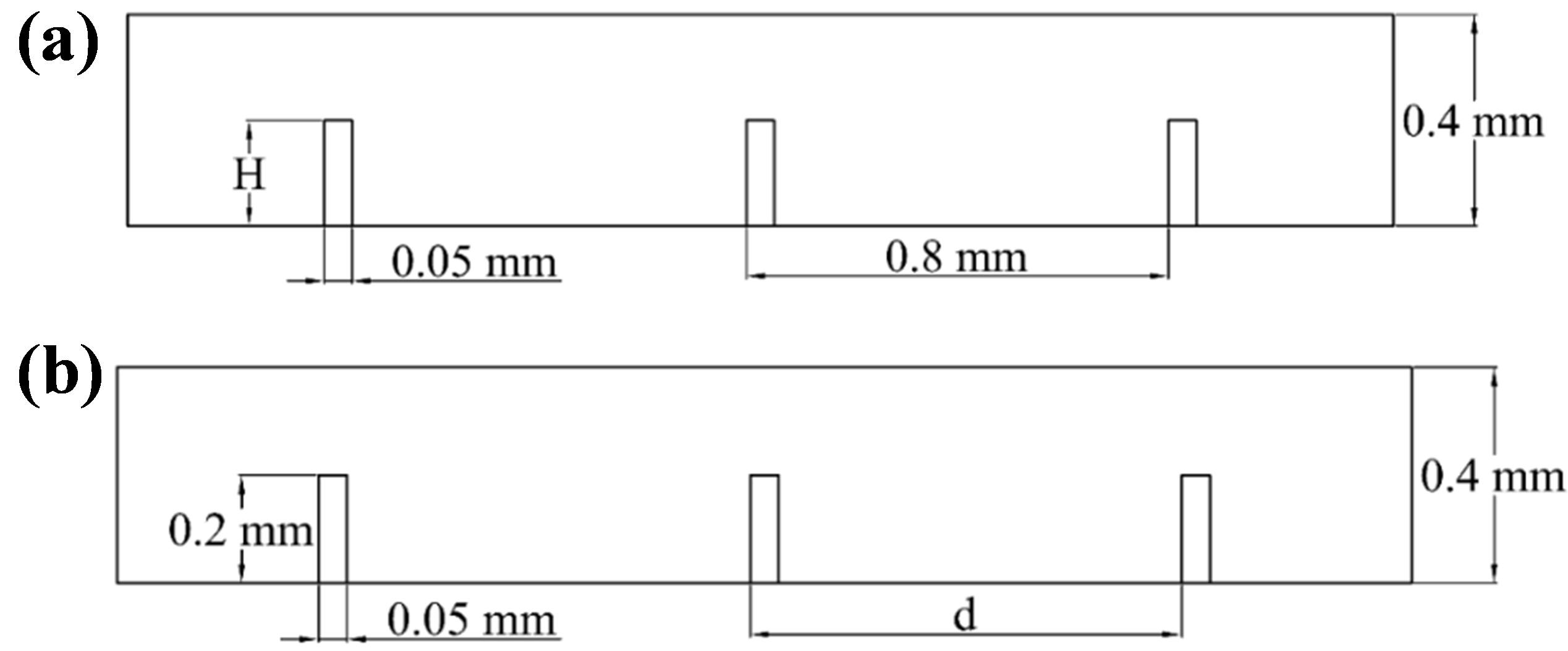


**Fig. S4.** (a) Key dimensions of sharp-edged microstructures with different heights; (b) Key dimensions of sharp-edged microstructures with different heights.

**Table S3.** HPQDs concentration distribution maps on the XOY plane for microchannels with spike microstructures at different heights.


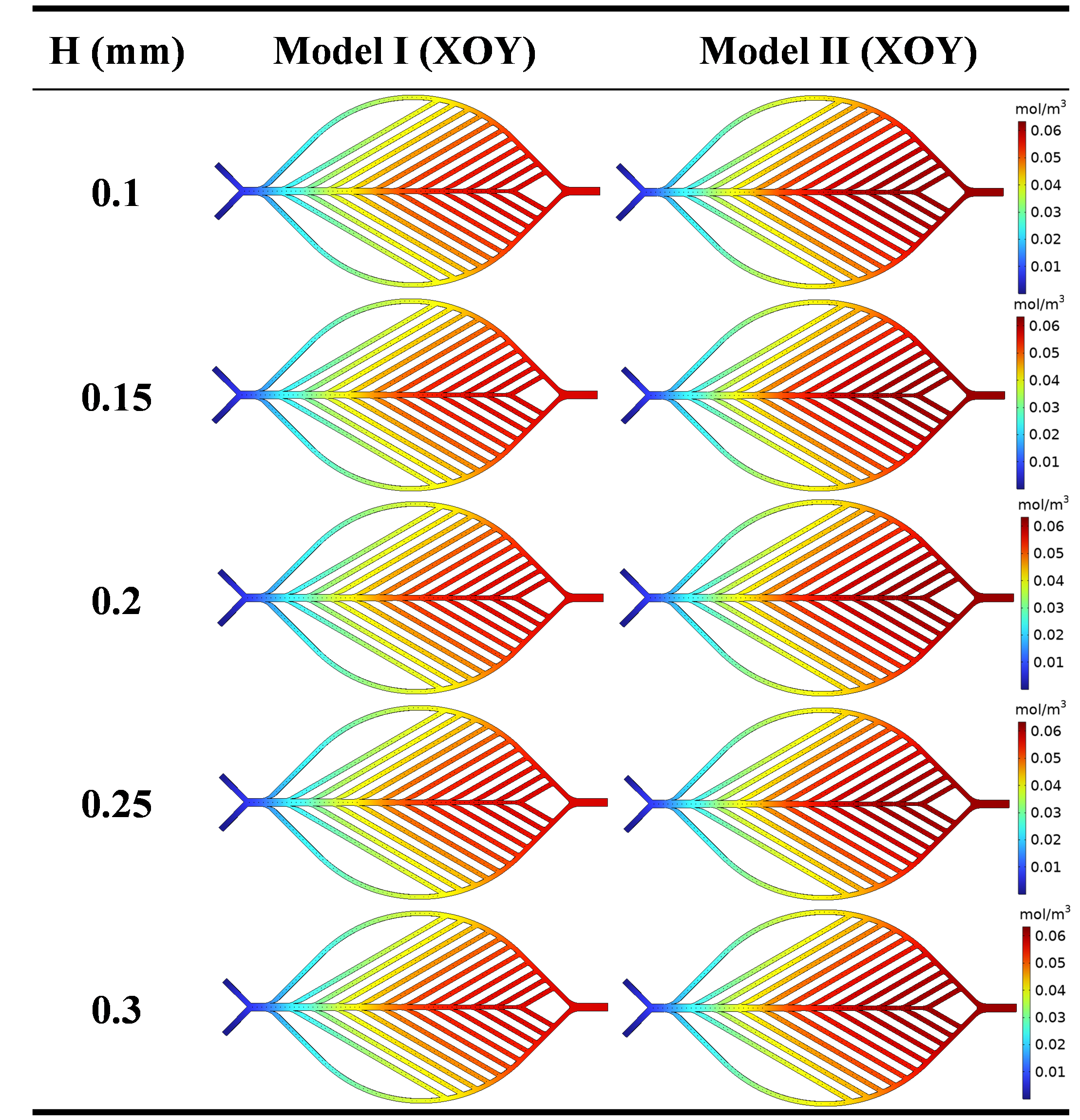


**Table S4.** HPQDs concentration distribution maps on the XOY plane for microchannels with elliptical microstructures at different heights.

.
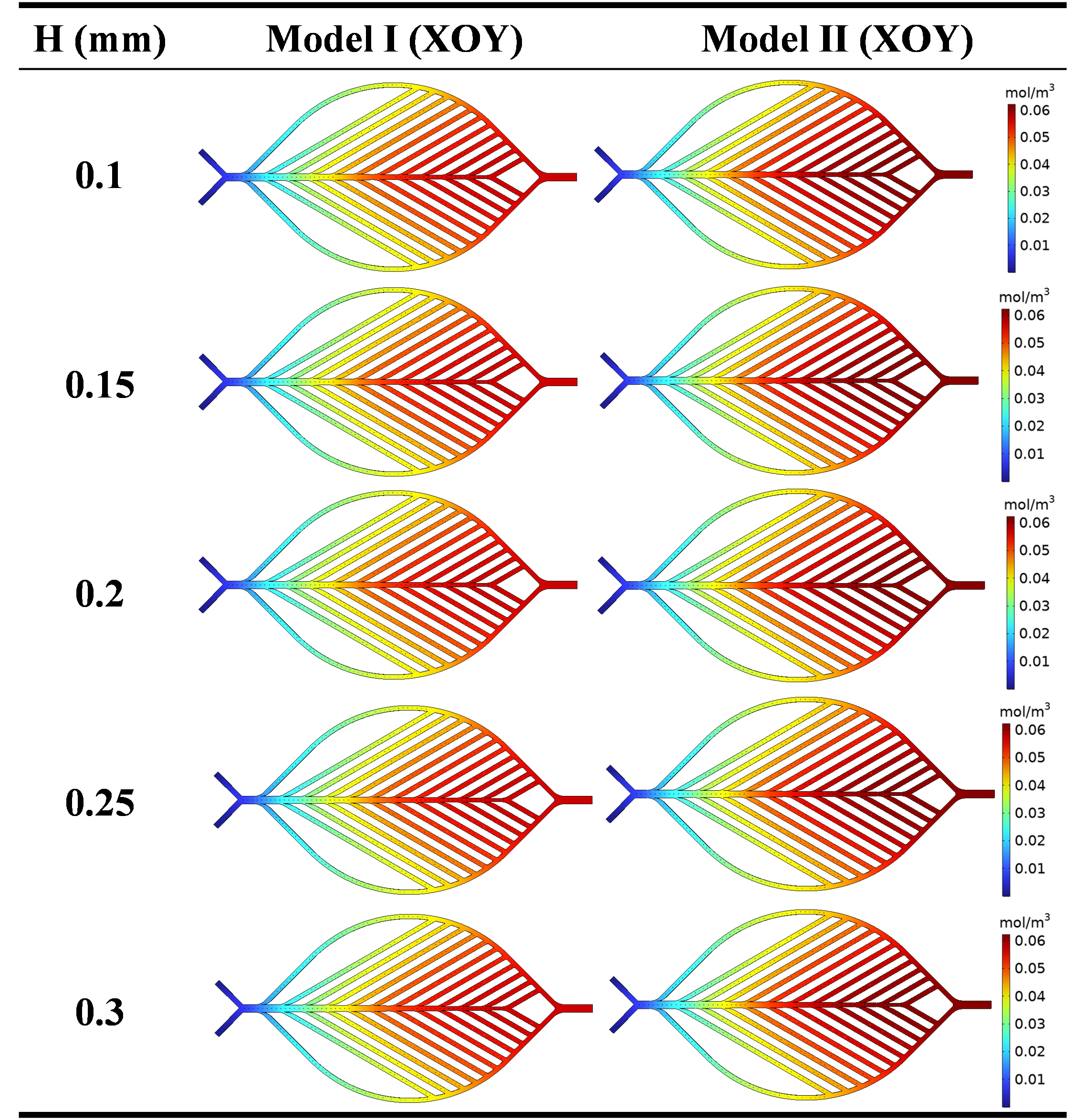


**Table S5.** Local streamline diagrams for microchannels with spike microstructures at different heights.


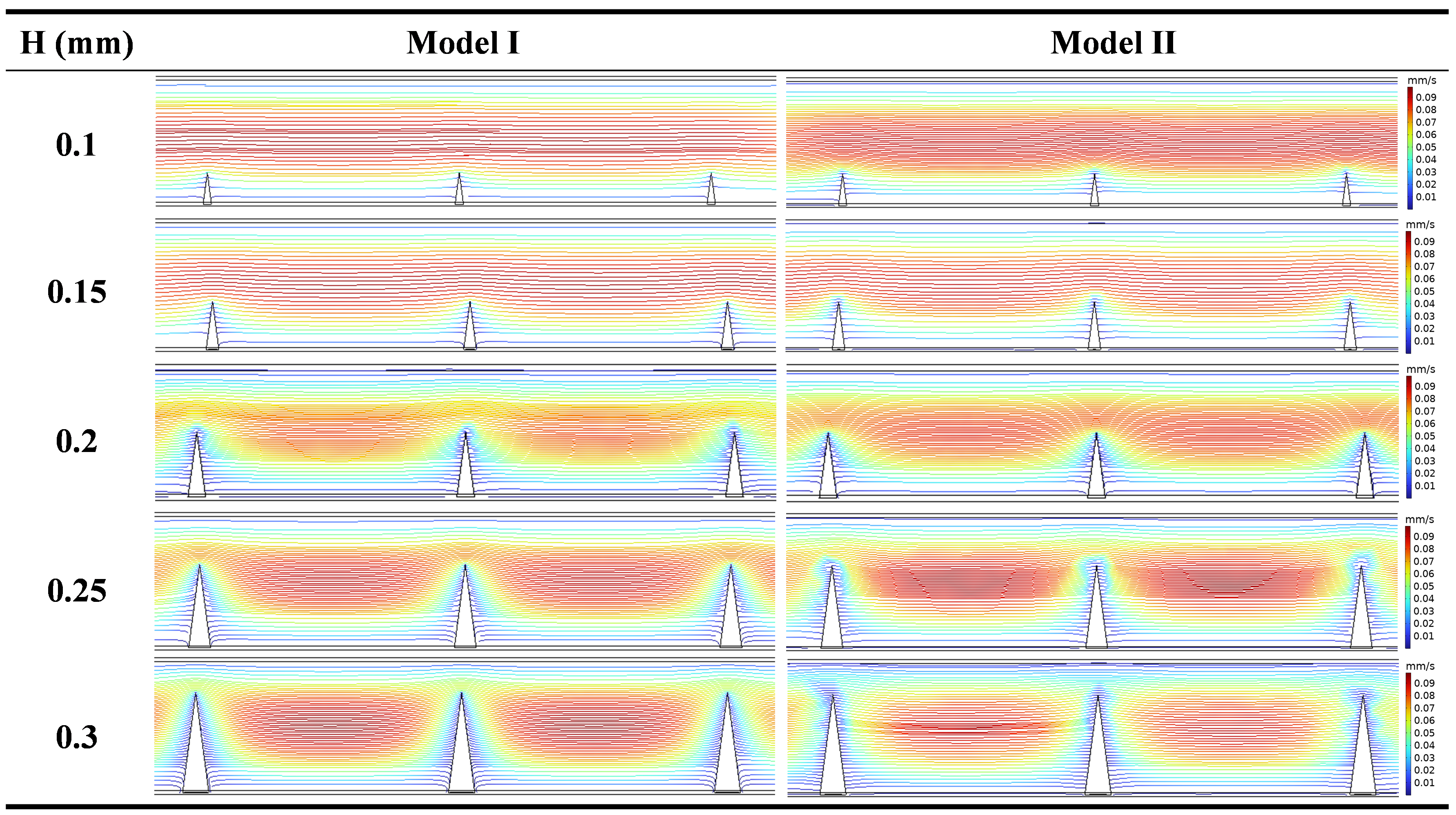


**Table S6.** Local streamline diagrams for microchannels with elliptical microstructures at different heights.


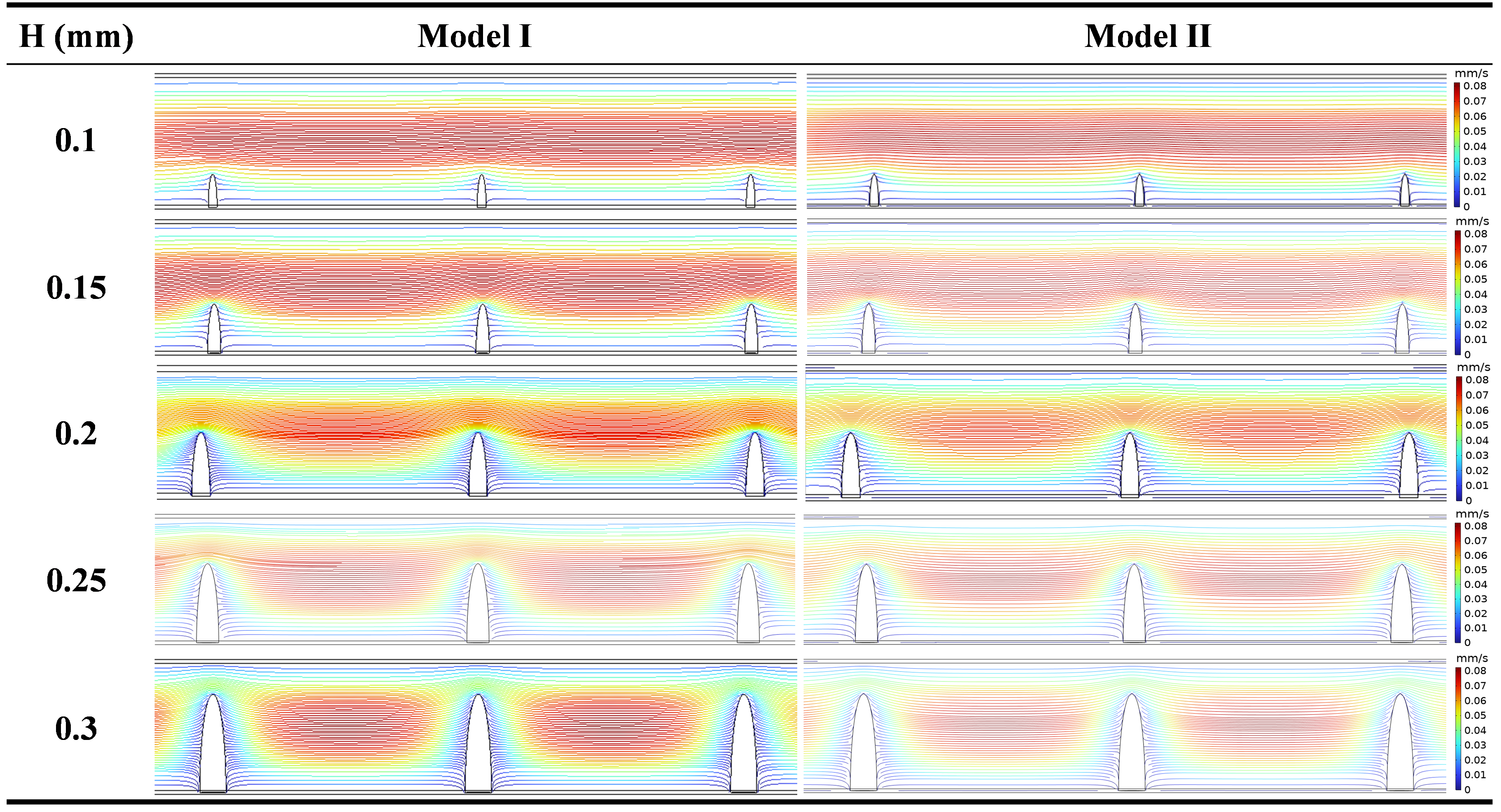


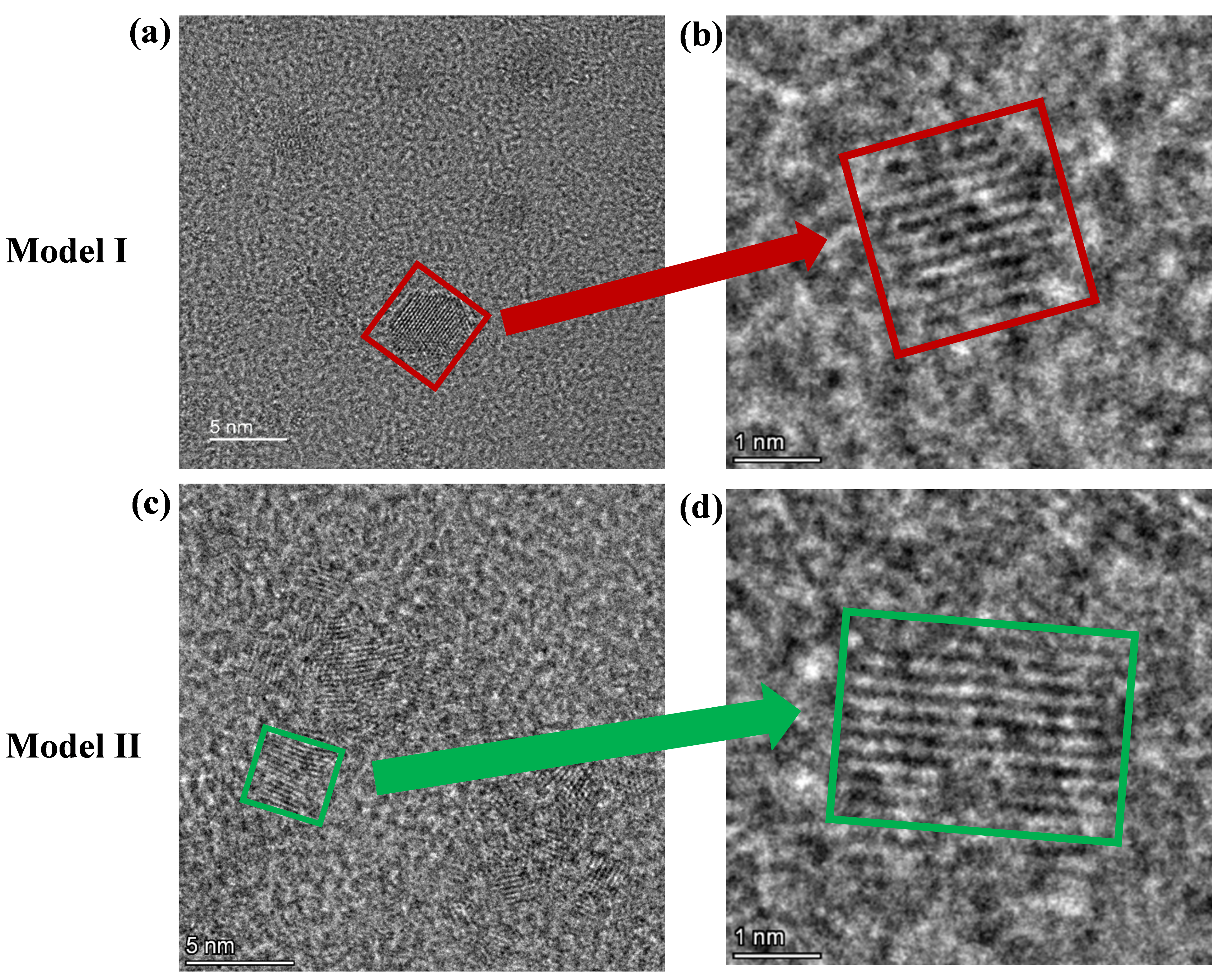


**Fig. S5.** HRTEM images of the HPQDs synthesized under different models: (a) 5 nm scale-bar image of Model I, (b) 1 nm scale-bar image of Model I, (c) 5 nm scale-bar image of Model II, and (d) 1 nm scale-bar image of Model II.


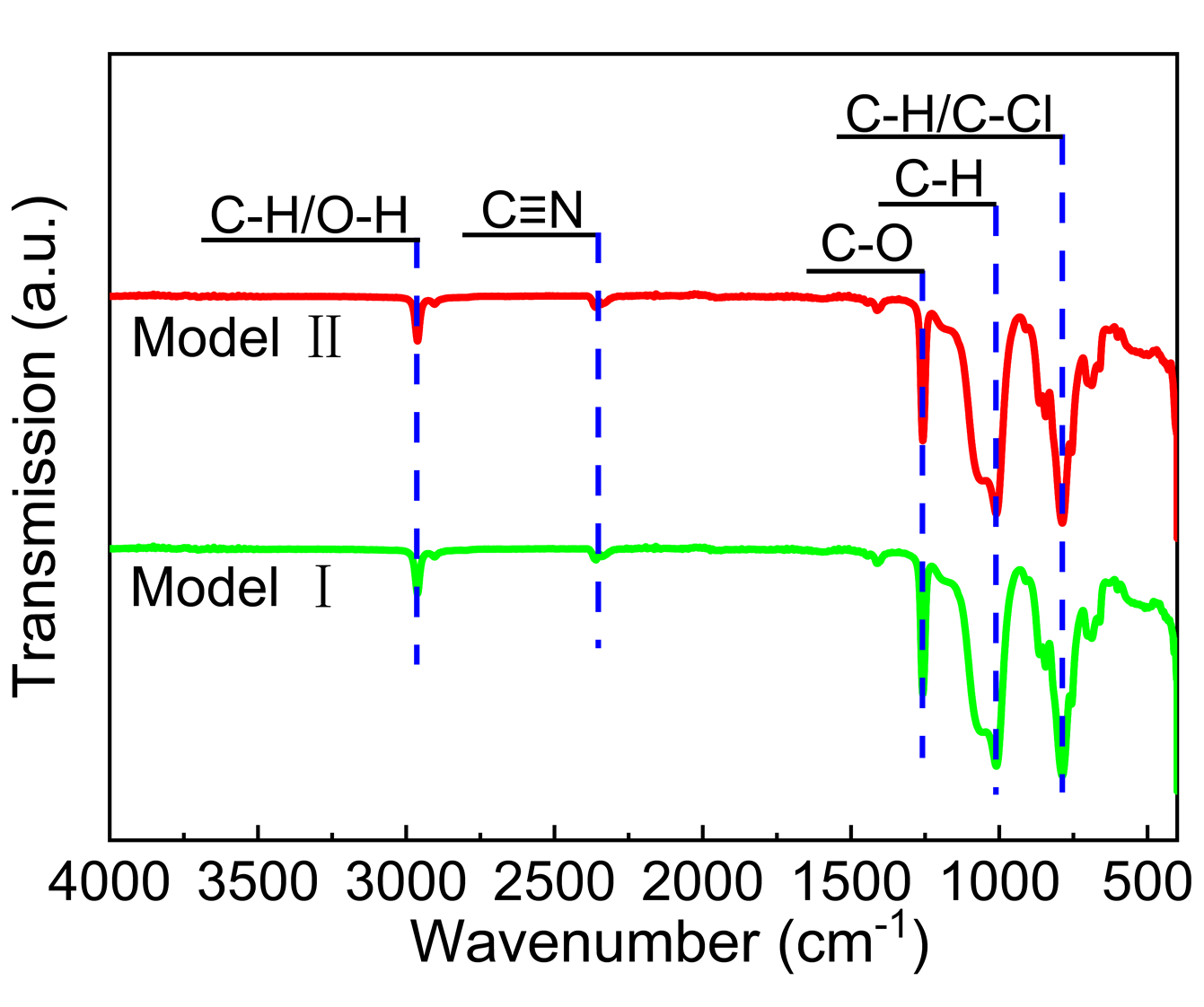


**Fig. S6.** FTIR of the HPQDs synthesized under Model I and Model II.

**Table S7.** Fitted lifetimes of the HPQDs synthesized under Model I and Model II.

|  | A_1_ | τ_1_ | A_2_ | τ_2_ | τ_avg._ |
| --- | --- | --- | --- | --- | --- |
| Model Ⅰ | 0.78 | 3.92 | 0.16 | 64.43 | 50.59 |
| Model Ⅱ | 0.68 | 23.34 | 0.19 | 100.91 | 65.78 |


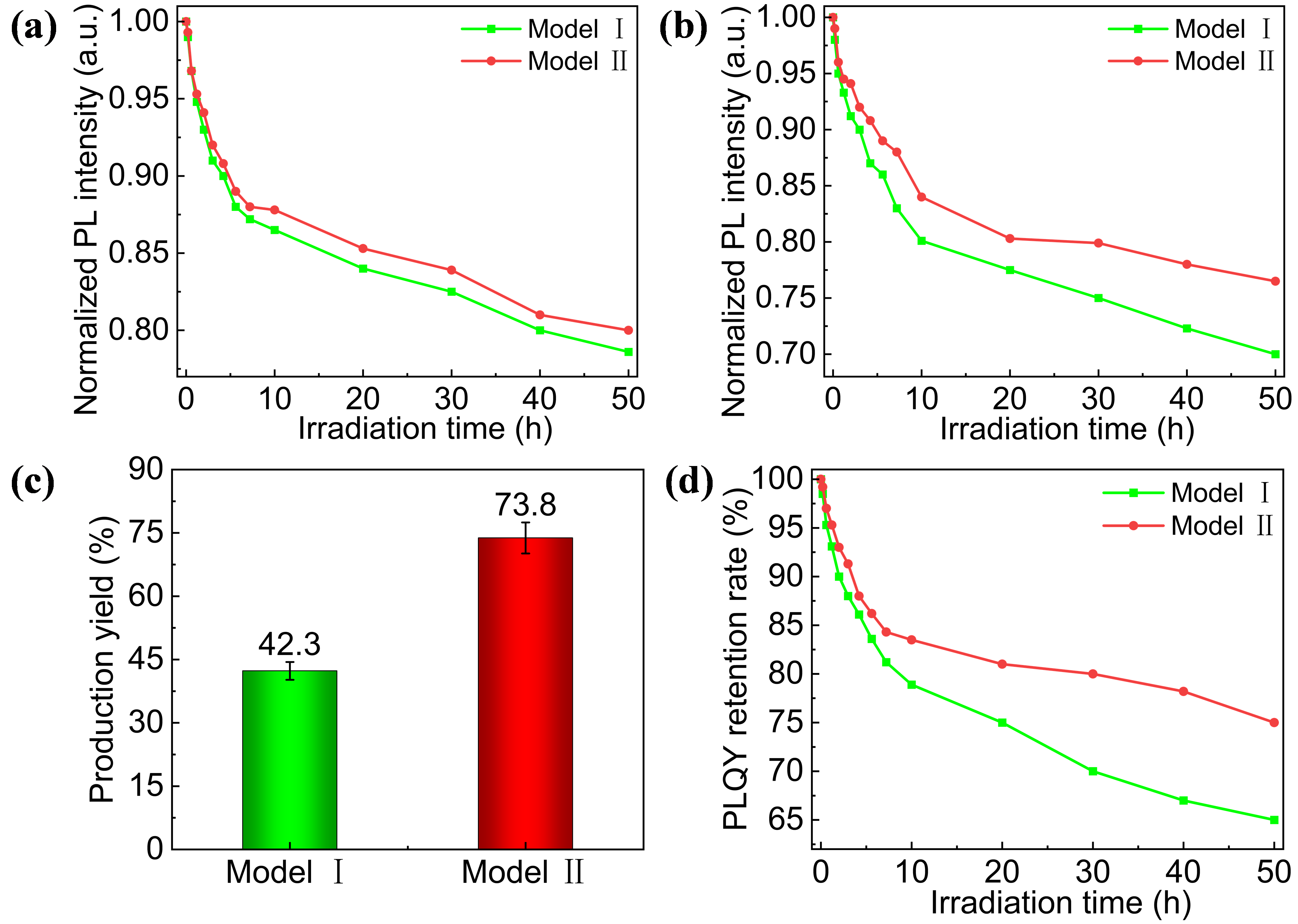


**Fig. S7.** (a) The photostability of the HPQDs synthesized under Model I and Model II. (b) The long-term stability of the HPQDs synthesized under Model I and Model II. (c) The production yield of the HPQDs synthesized under Model I and Model II. (d) The PLQY retention rate stability of the HPQDs synthesized under Model I and Model II.

**Table S8.** The FWHM of HPQDs excited with different wavelengths (360-460 nm) with Model I and Model II.

|  | **360 nm EX** | **365 nm EX** | **380 nm EX** | **400 nm EX** | **420 nm EX** | **440 nm EX** | **460 nm EX** |
| --- | --- | --- | --- | --- | --- | --- | --- |
| **Model I (nm)** | 27.8 ± 2.0 | 29.0 ± 1.3 | 30.7 ± 1.5 | 33.9 ± 1.2 | 38.8 ± 2.7 | 29.8 ± 3.0 | 45.9 ± 2.2 |
| **Model II**  **(nm)** | 23.6 ± 2.1 | 23.3 ± 1.0 | 23.6 ± 2.5 | 23.6 ± 2.4 | 23.7 ± 2.4 | 23.7 ± 3.0 | 23.6 ± 2.1 |
